# CCPG1 Is a Non-canonical Autophagy Cargo Receptor Essential for ER-Phagy and Pancreatic ER Proteostasis

**DOI:** 10.1016/j.devcel.2017.11.024

**Published:** 2018-01-22

**Authors:** Matthew D. Smith, Margaret E. Harley, Alain J. Kemp, Jimi Wills, Martin Lee, Mark Arends, Alex von Kriegsheim, Christian Behrends, Simon Wilkinson

**Affiliations:** 1 Edinburgh Cancer Research UK Centre, MRC Institute of Genetics and Molecular Medicine, University of Edinburgh, Edinburgh EH4 2XR, UK; 2 Munich Cluster for Systems Neurology (SyNergy), Ludwig-Maximilians-University Munich, München, Germany

**Keywords:** autophagy, ER-phagy, proteostasis, unfolded protein response, CCPG1, FIP200, Atg8, pancreas, tissue homeostasis

## Abstract

Mechanisms of selective autophagy of the ER, known as ER-phagy, require molecular delineation, particularly *in vivo*. It is unclear how these events control ER proteostasis and cellular health. Here, we identify cell-cycle progression gene 1 (CCPG1), an ER-resident protein with no known physiological role, as a non-canonical cargo receptor that directly binds to core autophagy proteins via an LIR motif to mammalian ATG8 proteins and, independently and via a discrete motif, to FIP200. These interactions facilitate ER-phagy. The *CCPG1* gene is inducible by the unfolded protein response and thus directly links ER stress to ER-phagy. *In vivo*, CCPG1 protects against ER luminal protein aggregation and consequent unfolded protein response hyperactivation and tissue injury of the exocrine pancreas. Thus, via identification of this autophagy protein, we describe an unexpected molecular mechanism of ER-phagy and provide evidence that this may be physiologically relevant in ER luminal proteostasis.

## Highlights

• CCPG1 is an ER stress-inducible ER-phagy cargo receptor in mammals
• CCPG1 binds directly to ATG8 proteins and FIP200 via distinct peptide motifs
• CCPG1 lysosomal degradation and ER-phagy both require these interactions
• CCPG1 maintains normal ER luminal proteostasis in pancreatic acinar cells *in vivo*

## Graphical Abstract

**Figure:**
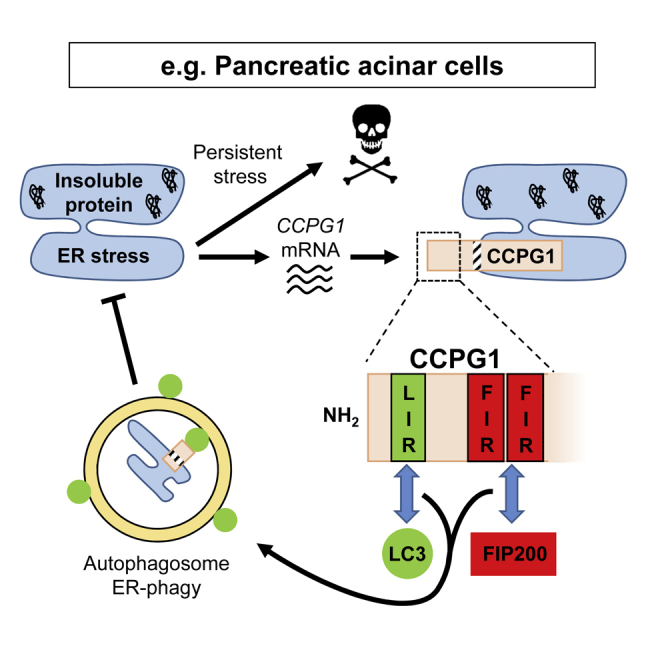

## Teaser

The mechanisms and physiological functions of ER autophagy (ER-phagy) are incompletely understood. Smith et al. show that the ER membrane-resident protein CCPG1 is an effector of ER stress responses, driving ER-phagy via interaction with ATG8 and FIP200. CCPG1 maintains ER luminal proteostasis within pancreatic acinar cells *in vivo*.

## Introduction

Macroautophagy (hereafter autophagy) is a conserved intracellular degradation mechanism that sequesters cytosolic cargoes into trafficking vesicles called autophagosomes, which then fuse with lysosomes (Ktistakis and Tooze, 2016). In mammalian cells, autophagy can be non-selective, catabolizing general cytosol (Lum et al., 2005). An important function of autophagy, however, is selective turnover of dysfunctional organelles (Khaminets et al., 2016), such as mitochondria (Wong and Holzbaur, 2014, Heo et al., 2015, Lazarou et al., 2015), peroxisomes (Kim et al., 2008, Deosaran et al., 2013), or lysosomes (Maejima et al., 2013), as well as degradation of ubiquitinated protein aggregates (Pankiv et al., 2007) or pathogens (Thurston et al., 2009, Zheng et al., 2009, Wild et al., 2011).

Mechanistically, autophagosomal membranes may form *de novo* or from the ER and/or mitochondria (Axe et al., 2008, Hayashi-Nishino et al., 2009, Hailey et al., 2010, Hamasaki et al., 2013), the ER-Golgi intermediate compartment (Ge et al., 2013), or plasma membrane- or endocytic pathway-derived vesicles (Ravikumar et al., 2010, Longatti et al., 2012). The ATG (autophagy) proteins cluster into several machineries required for engulfment (Ktistakis and Tooze, 2016). The ULK (uncoordinated 51-like kinase) complex is composed of a serine-threonine kinase (ULK1/2) and scaffold proteins ATG13, FIP200 (FAK interacting protein 200 kDa) (Ganley et al., 2009), and ATG101 (Hosokawa et al., 2009). ULK phosphorylates various ATG proteins and other autophagy players (Jung et al., 2009, Di Bartolomeo et al., 2010, Joo et al., 2011, Russell et al., 2013, Egan et al., 2015). The ULK complex, including FIP200, is recruited to sites of autophagosome biogenesis, preceding and facilitating the recruitment of other ATG assemblies (Ktistakis and Tooze, 2016). Ubiquitin-like ATG8 proteins of the LC3 and GABARAP subfamilies are recruited to these membranes via C-terminal lipidation (Slobodkin and Elazar, 2013). ATG8 family recruitment facilitates vesicle closure, as well as promoting post-engulfment steps (Nguyen et al., 2016, Tsuboyama et al., 2016). Recruitment of the ATG5-12/ATG16L1 complex (Gammoh et al., 2013) and ATG8 orthologs (Kraft et al., 2012) may also prolong ULK complex, including FIP200, retention at nascent autophagosomes. Other than its role within the ULK complex, no other autophagic functions for FIP200 have been identified.

Certain ATG proteins also participate in cargo recognition during selective autophagy. In yeast, selective autophagy receptors (SARs) are multi-functional Atg8, Atg11, and cargo-binding proteins (Farré and Subramani, 2016). Atg11 may also be important in recruiting active Atg1 (ULK ortholog) (Kamber et al., 2015, Torggler et al., 2016). The mammalian SAR equivalent is a cargo receptor (Khaminets et al., 2016). In mammals, the bridging of cargo to autophagy machinery occurs primarily via binding of ATG8 family members. There is no direct Atg11 ortholog in mammals, although FIP200 has some sequence similarity in its C terminus (Lin et al., 2013). ATG8 family binding occurs via a linear peptide motif known as the LIR, or LC3-interacting region (Pankiv et al., 2007, Ichimura et al., 2008).

It is plausible that autophagy could remodel the ER during homeostatic response pathways engaged by ER stress. The best-characterized of these is the unfolded protein response (UPR), which largely comprises transcriptional activation of pathways that resolve proteostatic defects within the ER lumen. The UPR is characterized by the activity of three signaling pathways emanating from ER-integral membrane sensor proteins, IRE1α, ATF6α, and PERK (Wang and Kaufman, 2016). When misfolded proteins accumulate in the ER lumen, these sensors trigger cascades that inhibit general translation while transcriptionally upregulating chaperones, oxidoreductases, ER-associated degradation (ERAD) proteins, and apoptotic mediators (Wang and Kaufman, 2016). High or sustained UPR signaling can lead to cell death and inflammation.

The UPR can stimulate generalized autophagic flux (Ogata et al., 2006) by transcriptional upregulation of *ATG* genes (Rouschop et al., 2010, B'Chir et al., 2013). It is not clear that this mechanism acts particularly in ER homeostasis; it constitutes modest global upregulation of autophagy. Nonetheless, ER-phagy, the autophagic sequestration of fragments of ER into autophagosomes, can occur in yeast (Lipatova and Segev, 2015) and mammalian cells (Tooze et al., 1990, Khaminets et al., 2015). Furthermore, some autophagy-deficient cell lineages exhibit expanded ER and ER stress signaling (Jia et al., 2011, Pengo et al., 2013). Notably, ER-phagy is distinct from the reported process of “ER-quality control-autophagy” (ERQC-autophagy), in which conformer mutants of proteins are cleared apparently via transfer from the ER into autophagosomes (Teckman and Perlmutter, 2000, Houck et al., 2014).

Recently, three mammalian membrane-anchored or transmembrane cargo receptors for ER-phagy have been discovered, FAM134B (Khaminets et al., 2015), RTN3 (Grumati et al., 2017), and Sec62 (Fumagalli et al., 2016). ER proteins that are SARs were also found in yeast (Mochida et al., 2015). FAM134B and RTN3 trim ER content via ER-phagy *in vitro*. Sec62 participates in ER-phagy during recovery from ER stress *in vitro*. Overall, it is not clear what mechanistic function ER-phagy pathways serve in homeostasis and cell health. Strikingly, there is little evidence for a role of ER-phagy specifically in proteostasis or for molecular mechanisms linking ER-phagy to the UPR. The physiological relevance of ER-phagy in animals also remains unclear. *In vivo*, wild-type *Fam134B* is required for the health of peripheral sensory neurons, which accumulate distended ER in mutant mice (Khaminets et al., 2015). Most tissues are unaffected by *Fam134B* mutation, pointing to the likely existence of undiscovered ER-phagy receptors.

ER function varies between different cell lineages *in vivo*. Professional secretory cells have expanded rough ER (rER), which facilitates high-level protein biosynthesis. The acinar cells of the adult exocrine pancreas secrete distinctive, heavily condensed granules of zymogens (inactive digestive enzymes) into the pancreatic ducts. A dynamically balanced UPR is critical for pancreatic acinar homeostasis (Lee et al., 2005). However, the role of autophagy here is unclear. Inhibition of general autophagy by knockout of *Atg5* or *Atg7* can lead to ER stress, dilation of ER, cessation of zymogen protein production, cell death, and inflammation (Antonucci et al., 2015, Diakopoulos et al., 2015), although not all reports wholly agree (Hashimoto et al., 2008, Antonucci et al., 2015, Diakopoulos et al., 2015). It is unlikely that the deleterious effects are related solely to ER homeostasis; however; the main pathology may be the energetic collapse of cell function associated with damaged mitochondria (Antonucci et al., 2015, Diakopoulos et al., 2015). These models do not experimentally dissect this from ER-phagy.

Here, we identify CCPG1 (cell-cycle progression gene 1) as a transmembrane, dual-affinity GABARAP/LC3- and FIP200-binding autophagy protein that partitions from the ER into the autophagy pathway. CCPG1 drives ER-phagy and ER remodeling downstream of the UPR in cultured cells. This requires interactions with both ATG protein classes, via two discrete interaction determinants, casting CCPG1 as a cargo receptor with a non-canonical mechanism of action. *In vivo*, CCPG1 maintains homeostasis of the pancreas by preventing accumulation of insoluble protein within the ER lumen, thus sustaining tissue health.

## Results

### Identification of CCPG1 as a Mammalian ATG8 Interactor

**Figure 1:**
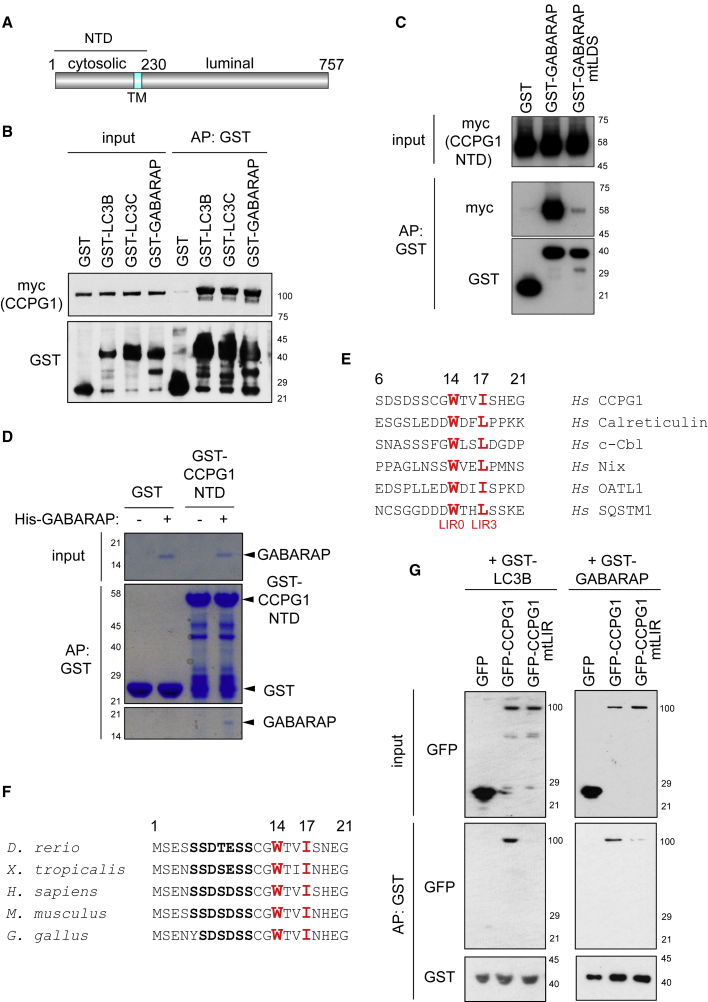
CCPG1 Is an LIR Motif-Containing Interactor of Human ATG8 Orthologs (A) Schematic of CCPG1 structure (NTD, N-terminal amino acids 1–230; TM, transmembrane anchor). (B) GST or GST fusions of ATG8 orthologs (LC3B, LC3C, and GABARAP) were used in affinity precipitation (AP) of transfected myc-CCPG1 from HEK293 cells. (C) GST or GST-GABARAP (mtLDS, LIR-docking site mutant) were used in AP of transfected myc-CCPG1 NTD from HEK293 cells. (D) Bacterially expressed GST or GST-CCPG1 NTD proteins were pre-purified on glutathione Sepharose beads and incubated with purified His-GABARAP before GST AP. (E) Human CCPG1 amino acids 6–21 aligned to tryptophan-containing LIR motifs from other human proteins. Important hydrophobic residues at LIR positions 0 and 3 are highlighted in red. (F) Alignment of the N terminus of human CCPG1 to other vertebrate CCPG1 proteins (human numbering). (G) HEK293 cells were transfected with the indicated GFP fusions of CCPG1, and AP of these fusions with GST-LC3B or GST-GABARAP was tested.

We performed an unbiased affinity-purification mass spectrometric screen for binding partners of GABARAP (Dunlop et al., 2014). Analysis of this dataset reveals a previously unknown interactor of GABARAP, so-called CCPG1. CCPG1 is a vertebrate-specific protein with no known physiological function. Organizationally, it has a cytosolic N-terminal region, a transmembrane domain that anchors it within the ER membrane, and an ER luminal C-terminal region (Kostenko et al., 2006) (Figure 1A). *CCPG1* was first identified as a human cDNA that blocked cell-cycle arrest in yeast (Edwards et al., 1997), but there is no evidence for a cell-cycle role in vertebrates. CCPG1 also binds Rho GTPase exchange factors, but the physiological significance of this is as yet undetermined (Kostenko et al., 2006).

To confirm CCPG1 function in autophagy, binding assays were firstly performed. GST fusions of the ATG8-family proteins LC3B, LC3C, and GABARAP could specifically affinity precipitate CCPG1 from cell lysates (Figure 1B). Additional assays were performed with the N-terminal region of CCPG1 (NTD), which lacks ER luminal sequence. These showed that GABARAP indeed bound to the cytosolic region of CCPG1, and that LIR-docking site mutants of GABARAP (mtLDS, Y49A, and L50A) had no affinity for CCPG1 (Figure 1C). These data implied that the cytosolic region of CCPG1 either contains an LIR motif or binds GABARAP indirectly through another LIR motif protein. Thus, an *in vitro* binding assay between purified, recombinant His-GABARAP and GST-CCPG1 NTD was performed, revealing the interaction to be direct (Figure 1D). Alignment of human CCPG1 with other human ATG8-binding proteins identified a putative LIR motif in the N terminus (Figure 1E), which is evolutionarily conserved (Figure 1F). Experimental confirmation of this LIR motif was obtained by mutation of key hydrophobic residues at positions 0 and 3 to alanine (mtLIR, W14A I17A), which inhibited binding to GABARAP and LC3B (Figure 1G).

These data show that CCPG1 interacts directly with ATG8 family proteins via a canonical LIR motif in the cytosolic N terminus of CCPG1.

### Identification of a Cytosolic Complex of CCPG1 and FIP200

**Figure 2:**
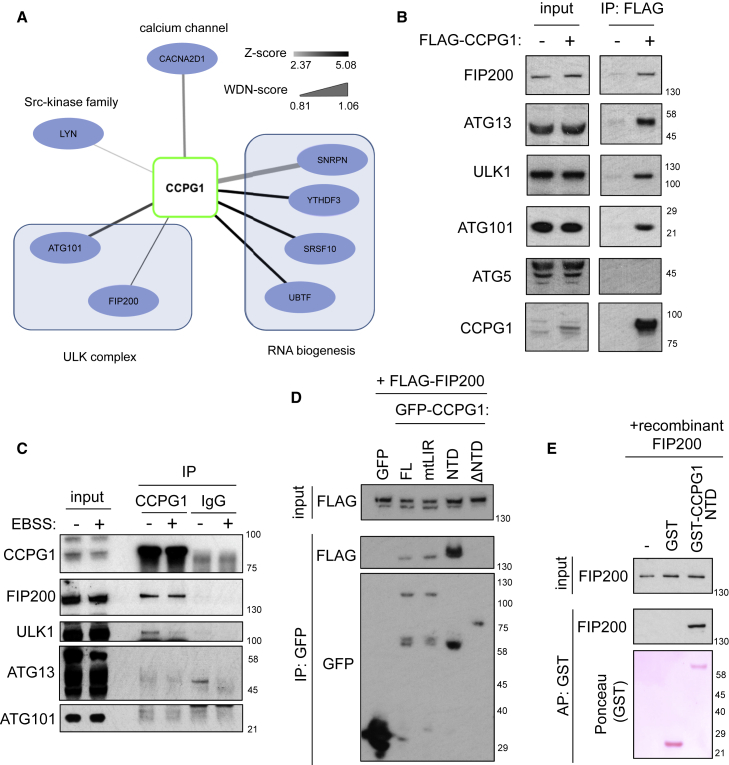
CCPG1 Is a FIP200-Interacting Protein (A) A549 NTAP (FLAG-HA)-CCPG1 cells were immunoprecipitated for tagged CCPG1 using anti-HA antibody and immunoprecipitates subjected to LC-MS/MS and CompPASS analysis (see the STAR Methods and Table S1). Interacting proteins at a cut-off of W_DN_ score 0.8 are shown here. (B) A549 cells stably expressing NTAP empty vector (−) or NTAP-CCPG1 (+) were immunoprecipitated for tagged CCPG1 with anti-FLAG beads and immunoblotted for indicated proteins. (C) A549 cells were EBSS starved or left untreated for 1 hr, prior to lysis and endogenous immunoprecipitation of CCPG1 and subsequent immunoblotting (IgG, negative control IgG). (D) HEK293 cells were transfected with FLAG-FIP200 and indicated variants of full-length (FL) GFP-CCPG1 (ΔNTD, amino acids 231–757). Immunoprecipitation was performed with GFP-Trap and immunoblotting performed with indicated antibodies. (E) Recombinant FIP200 was incubated with either glutathione Sepharose beads alone, or with pre-purified GST or GST-CCPG1 NTD bound beads. Affinity precipitation (AP) followed by immunoblotting was then performed to assess direct interaction. See also Figure S1 and Table S1.

Further identification of CCPG1-interacting partners was achieved by co-immunoprecipitation-tandem mass spectrometry (MS/MS)analysis of HA-CCPG1 immunoprecipitates from A549 lung cells (Table S1). ULK complex members, ATG101 and FIP200, were detected (Figure 2A). Overexpressed CCPG1 could interact with all ULK1 complex members in A549 cells, in contrast to ATG5, a component of a separate ATG machinery (Figure 2B). Furthermore, a constitutive co-immunoprecipitable complex of endogenous CCPG1 and FIP200 was detected strongly in the absence and presence of an autophagic stimulus of amino acid starvation (Earle’s buffered salt solution [EBSS], Figure 2C). A weaker interaction with ULK1, which was dissipated by EBSS, was also detected (Figure 2C), but no endogenous interaction was detected with ATG13 or ATG101 (Figure 2C). These data suggest a robust interaction of CCPG1 with FIP200. Validation of CCPG1 antisera for endogenous immunoblotting and immunoprecipitation was performed in A549 cells deleted for *CCPG1* (Figure S1A).

The interaction site within CCPG1 for FIP200 was mapped to the NTD, befitting the known cytosolic nature of FIP200 (Figure 2D). Furthermore, CCPG1 mtLIR retained interaction with FIP200, showing that CCPG1-FIP200 interaction is independent of CCPG1-ATG8 binding (Figure 2D). Finally, direct interaction between CCPG1 and FIP200 was demonstrated in a binding assay employing purified GST-CCPG1 NTD and purified, *in vitro* translated His-FIP200 (Figure 2E).

### A Linear Peptide Motif Mediates CCPG1-FIP200 Interaction

**Figure 3:**
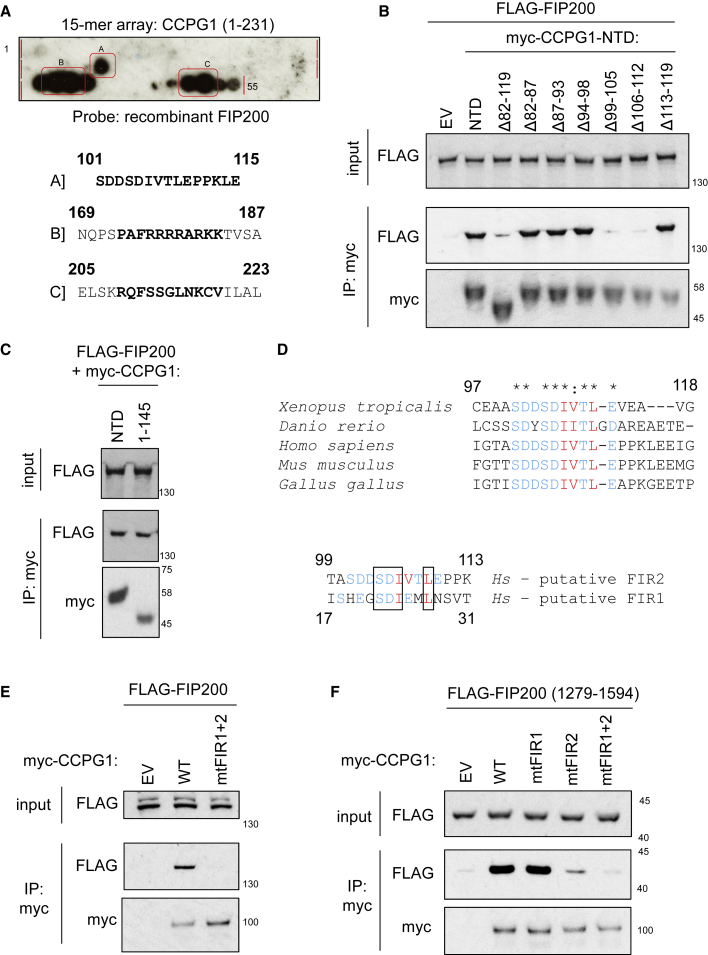
Identification of a Linear Peptide Motif in CCPG1 for Binding to FIP200 C-Terminal Region (A) A 15-mer peptide array (peptides 1–55) was probed with recombinant FIP200. Bound FIP200 was detected by indirect immunodetection. Peptide sequences corresponding to binding regions A–C are shown below the array. (B and C) HEK293 cells were transfected with FLAG-FIP200 and indicated myc-tagged deletions or truncations of CCPG1 NTD prior to anti-myc immunoprecipitation and immunoblotting (EV, empty vector). (D) Sequence alignment of the region from amino acids 97 to 118 of human CCPG1 against vertebrate orthologs (upper) or of regions amino acids 99–113 and 17–31 of human CCPG1 (lower). Conserved S/T and acidic residues are blue, hydrophobic residues are red. Asterisks indicate evolutionary conservation of residues. Black boxes indicate residues identical between FIR1 and FIR2. (E) HeLa Δ*CCPG1*-1 cells (Figure 5E) were transfected with FLAG-FIP200 and indicated variants of full-length myc-tagged CCPG1 (mtFIR1, S22A D23A I24A E25A; mtFIR2, S104A D105A I106A L109A), and immunoprecipitated on myc. (F) HEK293 cells were transfected with FLAG-FIP200 (1,279–1,594) and indicated variants of full-length myc-tagged CCPG1, and immunoprecipitated on myc. See also Figures S1 and S2.

The previous data show that FIP200 binds directly to the NTD of CCPG1 in an LIR-independent interaction. To address whether different linear peptide motif(s) in CCPG1 might bind FIP200, an immobilized peptide array spanning the N-terminal 231 residues of CCPG1 (Figure S1B) was probed with recombinant FIP200, highlighting three regions (Figure 3A, regions A–C). Co-immunoprecipitation assays using CCPG1 NTD mutants bearing deletions covering these regions were performed to determine if these sites bound FIP200 in solution. Region A covers amino acids (aa) 101–115. Deletion of aa 82–119 or smaller individual deletions of aa 99–105 or 107–112 ablated binding of CCPG1 with FIP200 (Figure 3B). No other deletions in the region of aa 82–119 had an impact upon binding. Thus, the peptide sequence corresponding to region A, between aa 99 and 112 was considered a candidate to contain a FIP200 interacting motif. Deletion of the C-terminal half of CCPG1 NTD (residue 146 onward) had no effect on FIP200 interaction (Figure 3C). Thus, we reasoned that regions B and C (aa 169–187 and 205–223, respectively), did not bind. In regard of region A, cross-vertebrate alignments revealed a conserved region between aa 101 and 110 (Figure 3D, upper alignment). In addition, a second peptide region within CCPG1, with sequence similarity to aa 101–110, was identified between aa 20 and 27 (Figure 3D, lower alignment). It was hypothesized that these regions contained “FIR” (FIP200 interacting region) motifs (the N-terminal motif being FIR1 and the distal motif FIR2). Indeed, mutation of four residues within each region to alanine ablated binding of CCPG1 with FIP200 (Figure 3E, mtFIR1+2). It was found that the FIR motif-containing regions, together, were required for binding to a small C-terminal domain fragment of FIP200 (aa 1,279–1,594). Interestingly, this domain encompasses the C-terminal Atg11 homology region of mammalian FIP200 (from aa 1,450 up to the C-terminal aa). The major contribution to binding was the originally identified motif from the peptide array, FIR2 (Figure 3F). The mutational tolerance of FIR2 was not defined. However, it was noted that the motif bore resemblance to yeast Atg11BR (Atg11 binding region) motifs, having a core of two hydrophobic residues surrounded by a field of negative charge and S/T residues (Figure S1C). The amino acid sequence in the region of FIR1 deviates from this consensus, perhaps consistent with its weaker binding.

FIR-dependent interaction of CCPG1 with FIP200 was also demonstrated in co-immunoprecipitation experiments from *Ulk1/2* double knockout (DKO) and *Atg13* null mouse embryonic fibroblasts, underscoring that the FIR motifs represent sites of direct contacts between CCPG1 and FIP200 (Figure S2A). Furthermore, a FIP200-binding-deficient mutant (mtFIR1+2) of full-length CCPG1 still binds GABARAP (Figure S2B). LIR and FIR mutants can thus dissect the functional importance of ATG8 versus FIP200 binding.

In summary, these data reveal two spatially distinct but sequence-related motifs in CCPG1, which mediate a direct interaction with FIP200, potentially akin to yeast SARs' mode of binding to Atg11.

### CCPG1 Traffics via the ER to Autophagosomes

**Figure 4:**
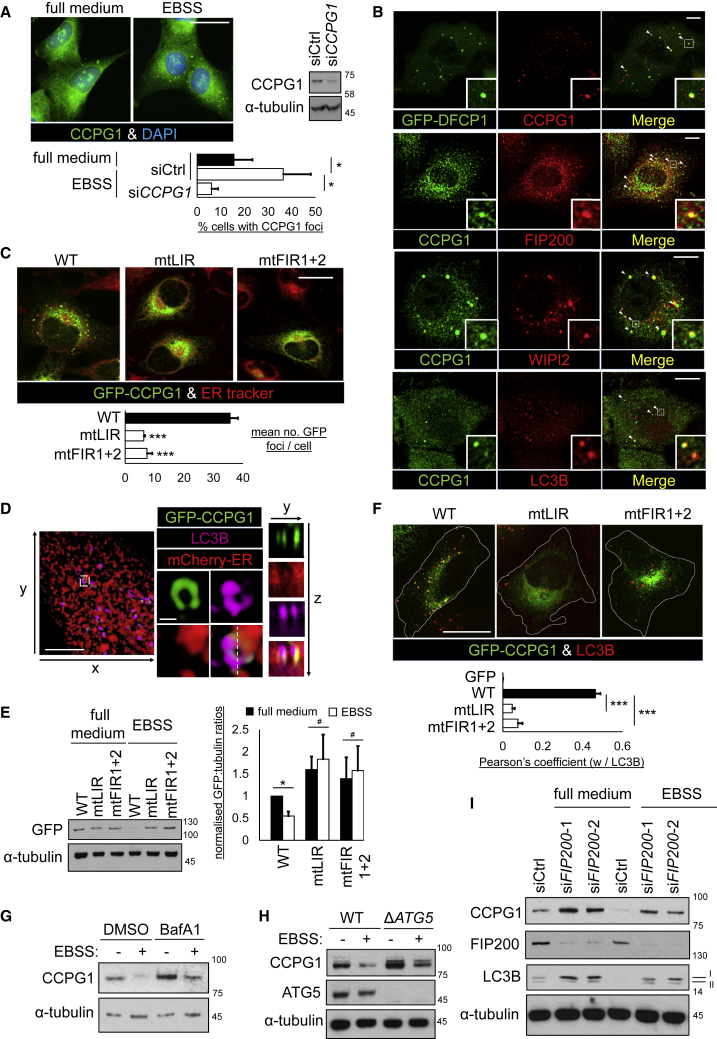
CCPG1 Is Recruited into Autophagosomes from the ER (A) A549 cells were transfected with siCtrl or si*CCPG1* and, at 24 hr post-transfection, either left untreated or starved for 1 hr in EBSS, then stained for endogenous CCPG1. Cells with CCPG1 foci were scored (n = 3, ± SEM, ^∗^p < 0.05, two-tailed paired sample t tests). Scale bar, 20 μm. (B) A549 or A549 GFP-DFCP1 cells were starved for 1 hr in EBSS and co-stained for endogenous CCPG1 and, for A549 cells, the indicated marker, then imaged by confocal microscopy. Arrowheads indicate co-localizing foci. Scale bars, 10 μm. (C) HeLa GFP-CCPG1 cells (wild-type [WT]) or indicated ATG8 (mtLIR) or FIP200 (mtFIR1+2) binding-deficient variants were starved, stained with ER tracker, and imaged by confocal microscopy. Automated quantification of GFP foci per cell was performed as described in the STAR Methods (n = 3, ± SEM, ^∗∗∗^p < 0.001, one-way ANOVA with Tukey's *post-hoc* test). Scale bar, 20 μm. (D) HeLa GFP-CCPG1 mCherry-ER cells were starved, stained for LC3B, and then imaged by 3D-SIM. Top left panel shows a reconstructed region of cell observed from above. The rightmost panels are zoomed images of the white boxed region. Lower panels show a cross-section along the white dashed line. Scale bars, 5 μm and 0.5 μm (zoomed). (E) HeLa GFP or GFP-CCPG1 cells, WT or indicated mutants, introduced in (D), were starved for 3 hr and blotted for GFP (left). Blots were quantified by densitometry for GFP:tubulin ratios (right) (n = 3, ± SEM, ^∗^p < 0.05, #not significant, two-tailed t test). (F) HeLa GFP or GFP-CCPG1 cells (WT or indicated mutants) were starved for 1 hr, co-stained for LC3B, and imaged by confocal microscopy. Pearson's coefficient for colocalization of GFP foci with LC3B was derived as described in the STAR Methods. White dashed lines indicate the outline of GFP-positive cells (n = 3, ± SEM, ^∗∗∗^p < 0.001, one-way ANOVA with Tukey's *post-hoc* test). Scale bar, 20 μm. (G) A549 cells were left untreated or starved for 4 hr in EBSS with or without 0.1 μM bafilomycin A1 (BafA1) and immunoblotted. (H) WT or Δ*ATG5* A549 clones were left untreated or starved for 4 hr in EBSS and immunoblotted. (I) A549 cells were transfected with siRNA for 48 hr and then starved and immunoblotted as shown (I and II indicate unlipidated and lipidated forms of LC3B, respectively). See also Figure S3.

To gain functional insight, the movement of CCPG1 between membrane compartments within cells was analyzed. Endogenous CCPG1 was localized under normal growth conditions to a perinuclear region, shown to be the ER (Figures 4A and S3A). A second population of either endogenous or exogenously expressed CCPG1 was detectable in the form of a small number of foci distributed around the periphery of the ER (Figures 4A and S3B). The abundance of these foci increased upon stimulation of autophagic flux with EBSS (Figures 4A and S3B). In the case of endogenous CCPG1 staining, RNAi-mediated knockdown of CCPG1 suppressed focus detection, validating the antiserum used (Figure 4A). The dynamics of CCPG1 compartmentalization was also followed by time-lapse fluorescence video-microscopy (Movie S1). Two differently mobile populations of GFP-CCPG1 were detected, correlating with those described above. In the perinuclear ER, a granular pattern of steady-state ER-resident CCPG1 was detected, which had low mobility. A second population of larger, more mobile CCPG1 foci was detected, which moved along peripheral ER tubules, many such foci remaining associated during the period of imaging.

In immunofluorescence staining experiments, it was then shown that the large foci colocalized with markers of multiple different stages of the autophagy pathway, including GFP-DFCP1, FIP200, WIPI2, and LC3B (Figures 4B and S3C). The large foci forming from the ER were thus interpreted as autophagosomal precursors or maturing vesicles, which contained CCPG1. Formation of CCPG1 foci was also observed to be dependent upon interaction with both mammalian ATG8s and FIP200, as shown by the failure of binding-deficient mutants to form puncta (Figure 4C). By super-resolution 3D-structured illumination microscopy (3D-SIM), the contiguous nature of CCPG1 and LC3B foci with the reticular network of the ER, as marked by mCherry-ER, was readily observable (Figure 4D).

Notably, some of the CCPG1 appears to be degraded by autophagy, further linking it to this trafficking pathway. Firstly, CCPG1 is lost upon starvation of cells in an LIR- and FIR-dependent manner (Figure 4E), consistent with a loss of localization of discrete CCPG1 puncta with LC3B foci (Figure 4F). In addition, CCPG1 is detected in a subset of LC3B-positive foci that are positive for STX17 (Figures S3D and S3E), but have no or relatively little signal for WIPI2, FIP200, or GFP-DFCP1 (Figure S3D), and is also detectable in LAMP2-positive lysosomes (Figure S3F) upon stimulation of autophagy. These imaging data are consistent with onward trafficking of some CCPG1 molecules from autophagosome formation sites at the ER to downstream stages of the pathway. In addition, CCPG1 levels are increased by blocking lysosome function with bafilomycin A1 (Figure 4G), CRISPR/Cas9-mediated deletion of *ATG5* (Figure 4H), or RNAi-mediated silencing of *FIP200* (Figure 4I).

Taken together, the above data show that CCPG1 is clustered from the ER membrane into degradation-competent autophagic vesicles, dependent upon discrete interactions with ATG8s (GABARAP/LC3) and FIP200.

### The UPR Drives CCPG1-Dependent ER-phagy Dependent upon ATG8 and FIP200 Binding

**Figure 5:**
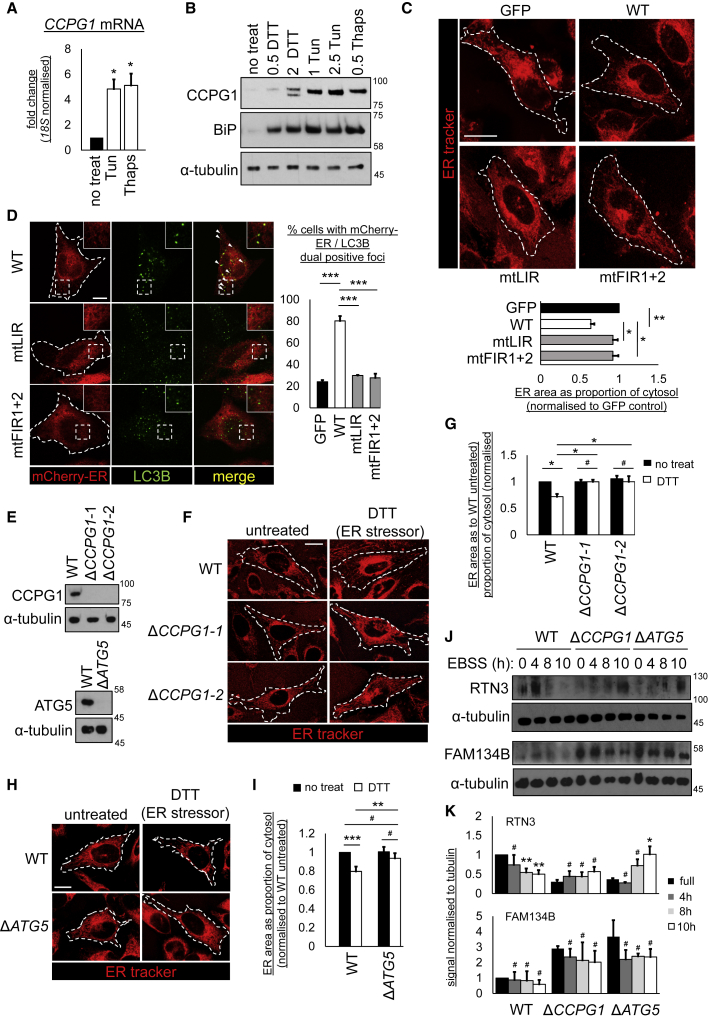
CCPG1 Is a UPR-Inducible Gene that Remodels the ER (A) A549 cells were treated with indicated ER stressors for 16 hr (Tun, tunicamycin, 2.5 μg/mL and Thaps, thapsigargin, 0.5 μM). qRT-PCR was performed for *CCPG1* (n = 3, ± SEM, ^∗^p < 0.05, one-way ANOVA followed by Tukey's *post-hoc* test). (B) HeLa cells were treated with indicated ER stressors (DTT, 0.5 or 2 mM, and Tun at 1 or 2.5 μg/mL, or Thaps at 0.5 μM) for 16 hr and then immunoblotted. (C) HeLa GFP-CCPG1 cells and variants were analyzed for ER peripheral morphology after ER tracker staining and confocal microscopy, as described in the STAR Methods. Values are given as area of ER in periphery as a proportion of cytosolic area. White dashed lines indicate the outline of GFP-positive cells (n = 3, ± SEM, ^∗^p < 0.05, ^∗∗^p < 0.01, one-way ANOVA with Tukey's *post-hoc* test). Scale bar, 20 μm. (D) HeLa GFP-CCPG1 cells and variants were transfected with mCherry-ER to mark ER membranes and immunostained for LC3B. mCherry-ER/LC3B double-positive foci-containing cells were scored by confocal microscopy, as described in the STAR Methods. White dashed lines indicate the outline of GFP-positive cells. Arrows indicate double-positive foci (n = 3, ± SEM, ^∗∗∗^p < 0.001, one-way ANOVA with Tukey's *post-hoc* test). Scale bar, 10 μm. (E–I) HeLa parental cells or CRISPR/Cas9 subclones deleted for *CCPG1* (Δ*CCPG1*-1 and Δ*CCPG1*-2) or *ATG5* (Δ*ATG5*) were (E) immunoblotted for CCPG1 or ATG5, or (F–I) analyzed for peripheral ER content after ER tracker staining as described above, either with or without 8 hr of 0.5 mM DTT treatment. White dashed lines indicate the outline of cells as determined by bright-field images (n = 3, ± SEM, ^∗∗∗^p < 0.001, ^∗∗^p < 0.01, ^∗^p < 0.05, #not significant, two-way ANOVA with Tukey's *post-hoc* test). Scale bars, 10 μm. (J and K) HeLa parental cells or deletants were starved with EBSS for indicated times and then immunoblotted. (K) Blots were quantified by densitometry for RTN3:tubulin (n = 4) or FAM134B:tubulin ratios (n = 3, ± SEM, ^∗∗^p < 0.01, ^∗^p < 0.05, ^#^not significant, two-tailed t tests). See also Figure S4.

It was hypothesized that *CCPG1* might be a UPR-regulated gene. Indeed, *CCPG1* mRNA (Figure 5A) and protein levels (Figure 5B) were induced by treatment with UPR inducers (DTT, tunicamycin, or thapsigargin) in A549 (Figure 5A) and HeLa (Figure 5B) cells. Focal sequestration of ER membrane into LC3B structures was observed at CCPG1-positive sites in the prior 3D-SIM experiments (Figure 4D). Thus, it was conjectured that CCPG1 induction might drive ER-phagy. Accordingly, we employed assays recently established for ER-phagy in mammalian cells (Khaminets et al., 2015). CCPG1 was expressed in HeLa cells to monitor the effect on ER morphology and distribution. While GFP-CCPG1 promoted reduction of peripheral ER content, GFP alone or autophagy-incompetent mtLIR or mtFIR1+2 mutants did not (Figure 5C). This effect of wild-type GFP-CCPG1 was only observed when the autophagy pathway was intact, as shown by ablation of the effect by RNAi targeting *ATG5* (Figure S4). Similarly, only wild-type CCPG1 was able to promote the colocalization of discrete foci of ER membrane, as marked by mCherry-ER puncta, with LC3B-positive autophagosomal puncta (Figure 5D). Deletion of endogenous *CCPG1* by CRISPR/Cas9 (Figure 5E) prevented the UPR inducer DTT from depleting peripheral ER (Figures 5F and 5G), as did deletion of endogenous *ATG5* (Figures 5E, 5H, and 5I). Finally, *CCPG1-* or *ATG5-*deleted HeLa cells retained greater amounts of the peripheral/tubular ER antigen RTN3 after stimulation of large-scale ER-phagy with EBSS, while demonstrating no differential retention of the perinuclear/sheet ER marker FAM134B. We interpret this to mean that CCPG1 can facilitate autophagic degradation of the peripheral ER, in line with the above imaging analyses (Figures 5J and 5K).

Taken together, these data show that *CCPG1* is a UPR-inducible gene, and its interaction with ATG8 orthologs and FIP200 can drive ER remodeling and ER-phagy, which occurs endogenously when the UPR and *CCPG1* transcription are activated by ER stress.

### CCPG1 Hypomorphic Mice Have a Pancreatic Proteostasis Phenotype

**Figure 6:**
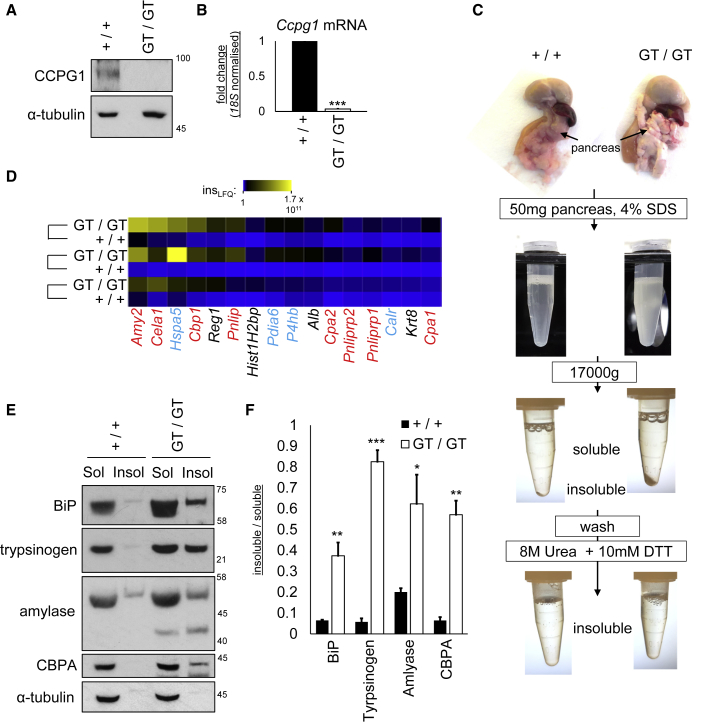
Defective Proteostasis in the Pancreas of *Ccpg1* Hypomorphic Mice (A and B) Whole pancreata from littermate 6-week-old WT (+/+) or *Ccpg1* hypomorphic (*GT*/*GT*) mice were immunoblotted for CCPG1 or subjected to RNA extraction and qRT-PCR for *Ccpg1* (n = 3 pairs, ± SEM, ^∗∗∗^p < 0.001, two-tailed t test). (C and D) Fifty mg of whole pancreata from littermate pairs of 6-week-old WT and *Ccpg1* hypomorphic mice were homogenized in SDS. Insoluble protein was pelleted, washed and extracted in 8 M urea +10 mM DTT. Pellet samples were normalized according to protein concentration in the soluble fraction and subjected to label-free LC-MS/MS quantification. A median absolute deviation analysis is presented as a heatmap here to show species changing significantly between pairs of mice (pairs joined by connecting brackets). Secretory enzymes are in red, ER luminal chaperones/oxidoreductases are in blue. (E and F) Detergent soluble and insoluble samples prepared as above were immunoblotted and ratios of insoluble to soluble protein species obtained via densitometry (n = 3 pairs, ± SEM, ^∗^p < 0.05, ^∗∗^p < 0.01, ^∗∗∗^p < 0.001, two-tailed t tests). See also Figure S5 and Table S2.

It is unclear why ER-phagy proteins trim the ER and how this contributes to homeostasis. Thus, it was hypothesized that the role of ER-phagy, via CCPG1 at least, might be revealed physiologically in tissues prone to ER stress, such as the exocrine pancreas. Thus, to investigate the *in vivo* function of CCPG1, a hypomorphic (gene-trapped, *Ccpg1*^GT^) mouse was generated by embryonic stem cell microinjection (Figure S5A). CCPG1 protein was undetectable in whole pancreatic extracts of homozygous mice (GT/GT, *Ccpg1*^GT/GT^), and mRNA abundance was reduced 100-fold (Figures 6A and 6B). Gross examination of pancreata of 6-week-old CCPG1-deficient mice revealed abnormalities, these tissues appearing whitened and opaque relative to control pancreata (Figure 6C, top). This phenomenon was unique to *Ccpg1* gene-trap homozygous mice (0/58 wild-type, 0/8 *Ccpg1*^GT/+^, 56/56 *Ccpg1*^GT/GT^ mice). This opaque mass could not be solubilized with strong detergent (Figure 6C). However, it could be pelleted and resolubilized in a urea-based buffer, showing it to be insoluble protein. Label-free quantification liquid chromatography (LC)- MS/MS was performed to analyze the material enriched in the insoluble fraction, using littermate-paired and sex-matched pairs of wild-type and CCPG1-deficient mice (Table S2; Figure 6D). Top-ranked protein species differing in solubility between the two genotypes were identified (Figure 6D). The majority of insoluble proteins was either secretory enzymes synthesized within the acinar ER (Amy2, amylase; *Cela1*, elastase 1; *Cbp1*, carboxypeptidase B1; *Pnlip* and *Pnliprp1* and *Pnliprp2*, lipase and related proteins; *Cpa2* and *Cpa1*, carboxypeptidase A) or ER luminal chaperones and oxidoreductases (*Hspa5,* BiP; *Pdia6* and *P4hb*, protein disulfide isomerases; *Calr*, Calreticulin). Immunoblotting confirmed repartitioning from the soluble to the insoluble fraction for BiP, amylase, and carboxypeptidase A, as well as trypsinogen (an abundant luminal enzyme that would be predicted to behave similarly) (Figures 6E and 6F). There were no significant increases in gene expression for the enzymes that would have confounded interpretation of these results (Figure S5B).

It was concluded that there is a proteostatic defect in *Ccpg1*^GT/GT^ exocrine pancreata, in acinar cells, consistent with the uniquely heavy demands for protein biosynthesis via the rER in this cell type. ER-synthesized enzymes and ER luminal chaperones accumulate in insoluble aggregates.

### CCPG1 Maintains ER Structure and Proteostasis in the Exocrine Pancreas

**Figure 7:**
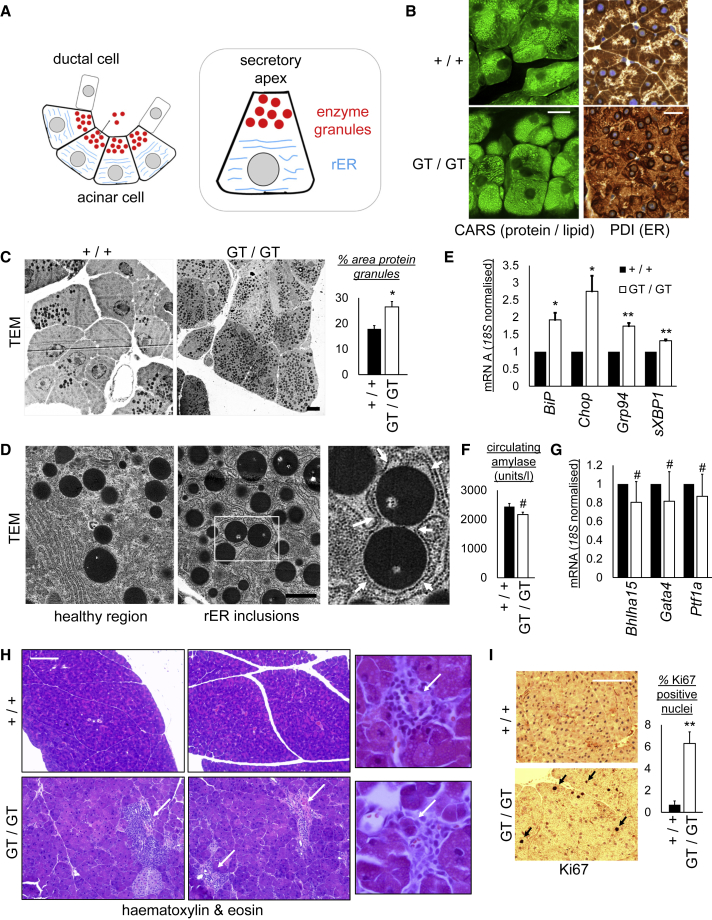
Loss of Cell Polarization and ER Homeostasis, and Consequent Tissue Injury, in *Ccpg1* Hypomorphic Exocrine Pancreata (A) The acinar unit of the exocrine pancreas. Polarized acinar cells secrete condensed enzyme (zymogen) granules into ducts from their apical stores. These enzymes are initially synthesized in the expansive rough ER (rER), which occupies the basolateral regions of the cell. (B) CARS imaging or immunohistochemical staining for the ER (protein disulfide isomerase, PDI) in pancreatic tissue from 6-week-old littermate WT (+/+) or *Ccpg1* hypomorphic (*GT*/*GT*) mice. Punctate CARS signals indicate protein or lipid inclusions. Scale bars, 20 μm. (C) Transmission electron microscopy (TEM) of pancreata from 6-week-old littermate pairs. Scale bar, 5 μm. Analysis of percent cytosolic area occupied by osmophilic protein granules was performed in ImageJ (n = 4 pairs, ± SEM, ^∗^p < 0.05, two-tailed t test). (D) High magnification TEM of a *Ccpg1* hypomorphic mouse reveals that the rER is distended and many supernumerary inclusions are in fact intracisternal granule-like structures (arrows in zoomed inset). Scale bar, 1 μm. (E) RNA from pancreata of 6-week-old littermate pairs of mice was assayed by qRT-PCR for levels of indicated UPR-inducible transcripts (n = 4 pairs, ± SEM, ^∗^p < 0.05, ^∗∗^p < 0.01, two-tailed t tests). (F) Plasma from pancreata of 32-day-old mice was analyzed for circulating amylase levels as described in the STAR Methods (n = 8, ± SEM, #not significant, two-tailed t tests). (G) RNA from pancreata of 6-week-old littermate pairs of mice was assayed by qRT-PCR for levels of indicated pancreatic acinar cell differentiation-associated transcripts (n = 4 pairs, ± SEM, ^#^not significant, two-tailed t tests). (H) H&E staining of representative samples from 40-week-old mice. Arrows highlight frequent inflammatory infiltrates observed in *Ccpg1* hypomorphic mice and, in zoomed panels, dead acinar cells often observed within the center of such infiltrates. Scale bar, 200 μm. (I) Immunohistochemical detection of proliferative cells (Ki67-positive nuclei) in formalin-fixed paraffin-embedded sections from 20-week-old littermate pairs of mice (n = 3 pairs, ± SEM, ^∗∗^ = p < 0.01, two-tailed t test). Arrows indicate Ki67-positive nuclei. Scale bar, 200 μm. See also Figures S5–S7.

To understand further the basis of the proteostatic defect in CCPG1-deficient mice, histological and ultrastructural analyses were performed. Ordinarily, acinar cells are highly polarized. The basolateral region of the cell contains rER. The cell apex stores clusters of dense enzyme granules (Figure 7A). Coherent anti-Stokes Raman spectroscopy (CARS) microscopy was used to detect dense lipid and protein clusters in optical (rather than cut) sections through intact segments of pancreata (Figure 7B). While wild-type mice have distinct polarization of protein granules, proteinaceous foci are distributed throughout the cell in CCPG1-deficient mice. Immunohistochemical analysis of ER also revealed loss of polarized distribution in CCPG1-deficient mice (Figure 7B). Transmission electron microscopy (TEM) showed that, while large protein granules were found exclusively at the apex of wild-type acinar cells, in CCPG1-deficient mice heterogeneously sized condensed protein granules or aggregates were observed throughout the cell, and the proportion of such material by area was greater than in wild-type mice (Figure 7C). High-resolution TEM showed that, in mutant cells, the rER was distended and the condensed or heterogeneously sized inclusions were trapped *within* the lumen (Figure 7D). These structures were interpreted as a late-stage manifestation of ER dysfunction; under such conditions, these inclusions are known to appear and to correlate with the accumulation of aggregation-prone ER enzymes and chaperones (Tooze et al., 1989, Tooze et al., 1990).

To determine if loss of ER proteostasis is detrimental to pancreatic health, transcriptional markers of elevated ER stress/UPR were assayed, including *BiP, Chop, Grp94*, and spliced *XBP1* (*sXBP1*), and found to be significantly upregulated in CCPG1-deficient pancreata by 6 weeks of age (Figure 7E). No significant reductions in plasma amylase levels (Figure 7F) or mRNA levels of key differentiation markers of the pancreatic exocrine lineage were observed in mutant pancreata (Figure 7G), suggesting no generalized defect in pancreatic exocrine function, including secretion. Mice have also been aged up to 48 weeks of age with no signs of frank morbidity or mortality. However, histological examination of the pancreata of older mice reveals numerous sporadic inflammatory infiltrates, particularly around necrotic cells and in the vicinity of ducts and blood vessels (Figure 7H). In addition, the manifestation of a significant fraction of proliferating, Ki67-positive cells in the ordinarily quiescent acinar cell population demonstrates compensatory proliferation, a known response to injury here (Figure 7I). Importantly, the loss of CCPG1 is not thought likely to affect the pancreas via a generalized developmental defect. The defective architecture of pancreatic acinar cells becomes apparent in young adult mice, not being apparent in 10-day-old mice or neonates (Figures S5C and S5D). Major organs, aside from the exocrine pancreas, are histologically normal in neonates and adult mice (Figures S5D and S6), with the exception of the adult gastric epithelium, wherein the chief cells, a polarized exocrine cell type with many architectural and molecular similarities to the pancreatic acinar cell, also display loss of polarity in histological sections (Figure S6, bottom center panel). It is likely that the defect here is analogous to that in pancreatic acinar cells, although this was not further analyzed in this study.

Considering the above data, loss of CCPG1-mediated ER proteostasis is concluded to result in unrestricted ER stress and tissue injury.

## Discussion

A model for CCPG1 function is shown in Figure S7. CCPG1 is an ER-resident transmembrane protein that is transcriptionally upregulated upon ER stress (UPR). CCPG1 residing in the ER membrane presents an N-terminal domain to the cytosol, which can interact directly via two separate linear peptide types (LIR and FIR motifs) with ATG8s and FIP200, respectively. Both interactions recruit CCPG1 to sites of autophagosome biogenesis at the ER and are required for upregulated CCPG1 to stimulate ER-phagy. *In vivo*, CCPG1 and, most likely, ER-phagy function (although other undiscovered roles of CCPG1 cannot be entirely excluded) maintains proteostasis within the lumen of the rER of the pancreatic acinar cell, protecting against elevated UPR signaling and tissue injury.

CCPG1 is identified here as an autophagy scaffold residing within the ER. CCPG1 fits some criteria of a canonical mammalian cargo receptor (Khaminets et al., 2016). It has an LIR motif that links the ER membrane to ATG8 orthologs on the immature autophagosome. However, non-canonically, CCPG1 also binds directly to FIP200 to promote ER-phagy. This observation poses several questions. Why does CCPG1 require FIP200, given that ATG8 binding is sufficient to impart selective autophagy function on canonical receptors? A recent study showed that yeast LIR motifs may actually bind non-Atg8 proteins, and may switch from these to Atg8 during selective autophagy, conferring directionality on the process (Fracchiolla et al., 2016). Perhaps CCPG1 may operate in a conceptually similar manner, but with the distinction of bearing spatially separated ATG-binding motifs, i.e., first binding FIP200 as a prerequisite for subsequent ATG8 interaction *in cellulo*. Alternatively, CCPG1 affinity for ATG8 orthologs on membranes within cells, rather than in solution, could be maximized by binding FIP200, potentially by facilitating clustering of CCPG1, although this remains speculative. It is notable that yeast SARs, the functional equivalent of cargo receptors, are defined by a molecular paradigm in which binding to Atg8 *and* Atg11 co-operate to facilitate selective autophagy. Here, Atg11 may mediate recruitment of active, or local activation of, the Atg1 kinase to cargo (Kamber et al., 2015, Torggler et al., 2016). Taken together with possible parallels between CCPG1 FIR motif interaction with FIP200 and the prototypical SAR-Atg11 interaction, it is plausible that CCPG1-mediated ER-phagy has mechanistic similarities with ER-phagy in yeast, which involves such dual Atg-binding SARs as Atg39 (Mochida et al., 2015). The above possibilities are not mutually exclusive and are consistent with a hierarchical model where FIP200 binds CCPG1 on the ER surface at pre-autophagosomal sites before substantial ATG8-family lipidation and generation of topologically distinct autophagosomal double membranes. This could resolve the apparent paradox that, in contrast to ATG8, FIP200 is commonly only found on the outer autophagosomal membrane of maturing autophagosomes, yet ER fragments are thought to be sequestered within the confines of the inner autophagosomal space.

Our observations also inform the question of whether FIP200's role in autophagy is solely via scaffolding the ULK complex to facilitate general autophagic flux. As well as the autophagy pathway, FIP200 has roles other than binding the ULK complex (Chen et al., 2016). The data presented here suggest for the first time that this may also be the case within the autophagy pathway.

Mammalian autophagy is upregulated at a non-specific level by ER stress via UPR-mediated induction of *ATG* genes. However, our data suggest a specific and direct link between the UPR signaling that emanates from the stressed ER and the selective process of ER-phagy. The identity of the transcription factor(s) within the different arms of the UPR cascade that drive *CCPG1* gene function remains to be conclusively determined. However, retrospective analysis of the dataset in a landmark single-cell transcriptomic analysis of the UPR suggests that *CCPG1* may be PERK responsive (Adamson et al., 2016). Also, the *CCPG1* promoter has been shown to bind the transcription factor MIST1 (Tian et al., 2010), which is tissue-specifically expressed in professional secretory cells such as pancreatic acinar cells. MIST1 expression is dependent upon the IRE1α-XBP1 arm of the UPR (Huh et al., 2010, Hess et al., 2011), thus providing a potential tissue-specific conduit for *CCPG1* upregulation by ER stress.

It is also possible that ER stress is not the only activating signal for CCPG1 activity. Some conventional cargo receptors have LIR motifs that are modified by phosphorylation at surrounding serine and threonine residues, modulating their ATG8-binding affinity (Wild et al., 2011, Khaminets et al., 2016). Similarly, the Atg11-binding regions of yeast SARs are prone to phosphorylation (Farré and Subramani, 2016). The LIR- and the FIP200-interacting peptide regions of CCPG1 are within serine- and threonine-rich sequences, suggesting potential phospho-regulation. Identification of such regulatory events might suggest other functions for CCPG1, i.e., outside of ER stress responses, or, alternatively, identify cytosolic signaling pathways that externally dictate the limits of ER-phagy capacity. The identity and purpose of such pathways remains speculative, but should be investigated in future studies.

*In vivo*, professional secretory tissues have been shown to require significant homeostatic regulation of the ER, via multiple transcriptional mechanisms, including foldase and chaperone expression, and ERAD. Such events are within the purview of the UPR. However, the role of ER-phagy has not been addressed. Here we show that CCPG1 is specifically involved in intraluminal ER proteostasis in the exocrine pancreas, rather than general secretion or exocrine pancreatic differentiation, placing selective autophagy firmly within the proteostatic toolkit of the UPR. It is likely that this reflects the ER-phagy role of CCPG1, although other as-yet-uncovered functions cannot be wholly excluded. This physiological role for ER-phagy would then also parallel the well-understood role for selective autophagy (aggrephagy) mechanisms in preventing protein aggregate accumulation within the cytosol. However, we consider it unlikely that CCPG1 has any role in cytosolic autophagy, even the degradation of mature zymogen granules. Zymophagy is only observed after treatment with disease-mimicking agents (Grasso et al., 2011) or prolonged starvation (Mizushima et al., 2004). Finally, while disease-associated mutants of ER-transiting proteins are cleared by ERQC-autophagy in very specific settings (Teckman and Perlmutter, 2000, Houck et al., 2014), CCPG1 provides the first evidence toward an ER luminal proteostatic function for autophagy in normal physiology.

Complete knockout of all autophagy function has previously been done in the exocrine pancreas by deletion of murine *Atg5* or *Atg7* (Hashimoto et al., 2008, Antonucci et al., 2015, Diakopoulos et al., 2015). The data from these studies are conflicting. However, a pancreatitis-like phenotype was frequently observed upon autophagy ablation, which did associate with elevated UPR signaling. However, in these studies the effects on tissue physiology were more severe than *Ccpg1* inhibition alone, as determined by this study, likely because of mitophagy and aggrephagy defects rather than ER-phagy defects (Antonucci et al., 2015, Diakopoulos et al., 2015). In fact, the severity of these phenotypes paradoxically results in arrest of transcription of ER-synthesized zymogens, which confounds the study of ER luminal proteostasis (Antonucci et al., 2015, Diakopoulos et al., 2015). Thus, *Ccpg1* hypomorphic mice provide a first model for examining what are potentially ER-specific autophagy events here.

Future investigations will determine whether translational strategies are devisable to manipulate CCPG1 function, and also whether CCPG1 has functions in pathways, other than autophagy, which might contribute to ER homeostasis *in vivo*. Speculatively, contexts where this manipulation of such functions of CCPG1 might be beneficial might be the amelioration of ER stress in pancreatic inflammatory states or, conversely, the enhancement of ER stress and elimination of malignant cells in pancreatic cancers.

## STAR★Methods

### Key Resources Table

**Table:** 

REAGENT or RESOURCE	SOURCE	IDENTIFIER
**Antibodies**		

Mouse mono anti-α-tubulin (clone DM1A) – IB	Sigma	T9026; RRID: AB_477593
Goat poly anti-Amylase (clone C20) – IB	Santa Cruz	sc-12821; RRID: AB_633871
Rabbit mono anti-ATG101 (clone E1Z4W) – IB	Cell Signaling Technologies	13492
Rabbit poly anti-ATG13 – IB	Sigma	SAB4200100; RRID: AB_10602787
Mouse mono anti-ATG13 – IB (Figure 2C only)	MBL	M183-3; RRID: AB_10796107
Rabbit poly anti-ATG5 – IB (Figure 2B)	Cell Signaling Technologies	2630; RRID: AB_2062340
Rabbit poly anti-ATG5 – IB (Figure 4I)	Sigma	A0731; RRID: AB_796188
Rabbit mono anti-BiP (clone C50B12) – IB	Cell Signaling Technologies	3177S; RRID: AB_2119845
Rabbit mono anti-Carboxypeptidase A (clone EPR12086) – IB	Abcam	ab173283
Rabbit poly anti-CCPG1 - IB, IP	Proteintech	13861-1-AP; RRID: AB_2074010
Rabbit poly anti-CCPG1 – IF Affinity-purified on N-term peptide	Eurogentec Double X programme	N/A
Rabbit anti-FAM134B - IB	Gift from Ivan Dikic, Goethe University, Frankfurt (Khaminets et al., 2015)	N/A
Mouse mono anti-FIP200 (clone 14E11.2) - IB (Figures 2C and S1A only)	Millipore	MABC128
Rabbit mono anti-FIP200 (clone D10D11) - IB, IF, Peptide Array	Cell Signaling Technologies	12436
Mouse mono anti-FLAG (clone M2) – IB	Sigma	F3165; RRID: AB_259529
Rabbit mono anti-GFP (clone D5.1) XP – IB	Cell Signaling Technologies	2956S; RRID: AB_1196615
Nanobody GFP (GFP-trap magnetic agarose beads) – IP	Chromotek	gtma-20; RRID: AB_2631358
Mouse mono anti-GST (clone GST-2) – IB	Sigma	G1160; RRID: AB_259845
Rat mono anti-HA (clone 3F10) – IF	Roche	11867423001; RRID: AB_10094468
Rabbit poly anti-Ki67 – IHC	Abcam	ab15580; RRID: AB_443209
Mouse mono anti-LAMP2 (clone H4B4) – IF	Abcam	ab25631; RRID: AB_470709
Rabbit mono anti-LC3B (clone D11) XP - IB, IF	Cell Signaling Technologies	3868; RRID: AB_2137707
Rabbit poly anti-Myc (anti-c-myc agarose conjugate) – IP	Sigma	A7470; RRID: AB_10109522
Mouse mono anti-Myc (clone 4A6) - IB (Figures 1B and 1C only)	Millipore	05-724; RRID: AB_11211891
Rat mono anti-Myc (clone JAC6) – IB	AbD Serotec	MCA1929; RRID: AB_322203
Rabbit mono anti-PDI (clone C81H6) – IHC	Cell Signaling Technologies	3501; RRID: AB_2156433
Rabbit poly anti-Rabbit IgG - IP control	Cell Signaling Technologies	2729; RRID: AB_2617119
Rabbit poly anti-RTN3 - IB	Millipore	ABN1723
Mouse mono anti-Syntaxin17 (clone 2F8) – IF	MBL	M212-3
Rabbit mono anti-TRAP alpha (clone EPR5603) – IB	Abcam	ab133238; RRID: AB_11157579
Mouse mono anti-Trypsinogen (clone D1) – IB	Santa Cruz	sc-137077; RRID: AB_2300318
Rabbit mono anti-ULK1 (clone D8H5) – IB	Cell Signaling Technologies	8054; RRID: AB_11178668
Mouse mono anti-ULK1 (clone F-4) - IB (Figure 2C only)	Santa Cruz	sc-390904
Mouse mono anti-WIPI2 (clone 2A2) – IF	AbD Serotec	MCA5780GA; RRID: AB_10845951
Anti-mouse IgG, HRP-linked antibody – IB	Cell Signaling Technologies	7076S; RRID: AB_330924
Anti-rabbit IgG, HRP-linked antibody – IB	Cell Signaling Technologies	7074S; RRID: AB_2099233
Anti-rat IgG, HRP-linked antibody – IB	Cell Signaling Technologies	7077S; RRID: AB_10694715
Goat anti-mouse IgG H+L AlexaFluor 488 – IF	ThermoFisher Scientific	A11001; RRID: AB_2534069
Goat anti-mouse IgG H+L AlexaFluor 594 – IF	ThermoFisher Scientific	A11005; RRID: AB_141372
Goat anti-mouse IgG H+L AlexaFluor 647 – IF	ThermoFisher Scientific	A21235; RRID: AB_141693
Goat anti-rabbit IgG H+L AlexaFluor 488 – IF	ThermoFisher Scientific	A11034; RRID: AB_2576217
Goat anti-rabbit IgG H+L AlexaFluor 594 – IF	ThermoFisher Scientific	A11012; RRID: AB_141359
Goat anti-rabbit IgG H+L AlexaFluor 647 – IF	ThermoFisher Scientific	A32733; RRID: AB_2633282
Goat anti-rat IgG H+L AlexaFluor 488 – IF	ThermoFisher Scientific	A11006; RRID: AB_2534074
Goat anti-rat IgG H+L AlexaFluor 594 – IF	ThermoFisher Scientific	A11007; RRID: AB_10561522

**Bacterial and Virus Strains**		

Rosetta II (DE3) cells	Novagen	71400-3
Library efficiency DH5α competent cells	Invitrogen	18263012

**Chemicals, Peptides, and Recombinant Proteins**		

35mm glass bottom dishes	World Precision Instruments	FD35-100
Anti-HA-agarose	Sigma	A7470
Bafilomycin A1	Sigma	B-1793
CCPG1 15-mer peptide array (amino acids 1-230)	JPT Peptide Technologies GmbH	Custom Order
cOmplete Protease Inhibitor Cocktail	Roche	11140920
Dako fluorescent mounting medium	Dako	S3023
DAPI	Sigma	D9542
DTT	Sigma	43815
EBSS	Sigma	E2888
ECL prime	Amersham	RPN2232
EDTA-free cOmplete Protease Inhibitor Cocktail	Roche	4693159001
EM grade gluteraldehyde	Sigma	G5882
ER Tracker Red	Molecular Probes	E34250
Fluoroshield mounting medium	Sigma	F6182
G418	Formedium	G418S
GluC	Promega	V165A
Glutathione Sepharose 4B beads	GE Healthcare	17-0756-01
High precision cover-glass	Zeiss	474030-9000-000
Hygromycin	Millipore	400052
IPTG	Fisher Scientific	BP1755-10
Lipofectamine 2000	Invitrogen	11668-019
Mass spec grade Trypsin	Promega	V5280
Oligofectamine	Life Technologies	12252-011
Puromycin	Fisher Scientific	BPE2956-100
RNAse-free water	Gibco	15230-089
Trizol	Ambion	15596026
Tunicamycin	Sigma	T7765
HA peptide	Sigma	I2149
Recombinant His-FIP200	Gift from Noor Gammoh, University of Edinburgh (Gammoh et al., 2013)	N/A

**Critical Commercial Assays**		

0.45 μm spin filter	Millipore	20-218
Bond Polymer Refine Detection	Leica	DS9800
Qiagen RNeasy kit	Qiagen	74106
QIAshredder	Qiagen	79654
qScript cDNA SuperMix	Quanta Biosciences	95048
DyNAmo HS SYBR Green qPCR Kit	ThermoFisher Scientific	F410L

**Experimental Models: Cell Lines**		

Human: Human Embryonic Kidney-293FT	Clontech	N/A
Human: HeLa female cervical carcinoma-EcoR	Gift from Ken Parkinson, Beatson Institute, Glasgow	N/A
Human: HeLa cervical carcinoma-EcoR Δ*ATG5*	This study	N/A
Human: HeLa cervical carcinoma-EcoR Δ*CCPG1*	This study	N/A
Human: HeLa-TetOff GFP	This study	N/A
Human: HeLa-TetOff GFP-CCPG1	This study	N/A
Human: HeLa-TetOff GFP-CCPG1 mtLIR	This study	N/A
Human: HeLa-TetOff GFP CCPG1 mtFIR1+2	This study	N/A
Human: A549 male lung cell carcinoma	Gift from Chris Marshall, ICR, London	N/A
Human: A549 lung cell carcinoma Δ*ATG5*	(Newman et al., 2017)	N/A
Human: A549 lung cell carcinoma Δ*CCPG1* clone 1	This study	N/A
Human: A549 lung cell carcinoma Δ*CCPG1* clone 2	This study	N/A
Human: A549 lung cell carcinoma stably expressing NTAP-CCPG1	This study	N/A
Human: A549 lung cell carcinoma stably expressing NTAP-CCPG1 mCherry-ER (KDEL)	This study	N/A
Human: A549 lung cell carcinoma stably expressing NTAP-CCPG1 GFP-DFCP1	This study	N/A
Human: Phoenix-Eco	Gift from Kevin Ryan, Beatson Institute, Glasgow	N/A
Mouse: mouse embryonic fibroblast (MEF) *Atg13*^*-/-*^	Gift from Noor Gammoh, IGMM, Edinburgh (Gammoh et al., 2013)	N/A
Mouse: mouse embryonic fibroblast (MEF) *Ulk1/2*^*-/-*^	Gift from Noor Gammoh, IGMM, Edinburgh (Gammoh et al., 2013)	N/A

**Experimental Models: Organisms/Strains**		

Male ES cells: Ccpg1^tm1a(EUCOMM)Hmgu^	EUCOMM	Clone ID: HEPD0725_5_F09
Male and Female Mice: C57/BL6N-Ccpg1^tm1a(EUCOMM)Hmgu^	This paper	MGI:5000356

**Oligonucleotides**		

Primers for qRT	See Table S3	N/A
siRNA oligonucleotides	See Table S3	N/A

**Recombinant DNA**		

gRNA vector	A gift from George Church, Harvard, USA (Mali et al., 2013)	Addgene plasmid # 41824
gRNA Atg5-2 21bp of target: TCTAAGGATGCAATTGAAGCC	This paper	N/A
gRNA CCPG1-3 19bp of target: TCTAACTTAGGTGGCTCAA	This paper	N/A
pDONR223 CCPG1 Human CCPG1 1-757	This paper	N/A
pDONR223 CCPG1 mtFIR1 S22A D23A I24A E25A Human CCPG1 1-757	This paper	N/A
pDONR223 CCPG1 mtFIR2 S104A D105A I106A L109A Human CCPG1 1-757	This paper	N/A
pDONR223 CCPG1 mtFIR1+2 S22A D23A I24A E25A S104A D105A I106A L109A Human CCPG1 1-757	This paper	N/A
pDONR223 CCPG1 mtLIR W14A I17A Human CCPG1 1-757	This paper	N/A
pDONR223 CCPG1 mtLIR1 + mtFIR1+2 W14A I17A S22A D23A I24A E25A S104A D105A I106A L109A Human CCPG1 1-757	This paper	N/A
pDONR223 CCPG1 NTD Human CCPG1 1-230	This paper	N/A
pDONR223 CCPG1 ΔNTD Human CCPG1 231-757	This paper	N/A
pDONR223 CCPG1 NTD Human CCPG1 1-230 with internal deletions or truncated from C-terminus, as indicated in main text	This paper	N/A
pDONR223 EV	Invitrogen	https://www.addgene.org/vector-database/2395/
pDONR223 hFIP200 Human FIP200 1279-1594	This paper	N/A
pDONR223 GABARAP mtLDS Y49A L50A	(Behrends et al., 2010)	N/A
pDONR223 mCherry-ER (KDEL)	This paper	N/A
pDEST 15	Invitrogen	Cat # 11802014
pDEST15-GST (empty vector)	(Newman et al., 2012)	N/A
pDEST15-GST-GABARAP	(Newman et al., 2012)	N/A
pDEST15-GST-LC3B	(Newman et al., 2012)	N/A
pDEST15-GST-LC3C	(Newman et al., 2012)	N/A
pBabe BSD mCherry DEST	This paper	N/A
pdcDNA 6x myc DEST	Created by F. Van Roy and B. Janssens, Ghent University, Belgium	BCCM plasmid #LMBP 7212
pdcDNA FLAG DEST	Created by F. Van Roy and B. Janssens, Ghent University, Belgium	BCCM plasmid #LMBP 4704
pEGFP C1 DEST	This paper	N/A
pmCherry-C1-DEST	This paper	N/A
pREV-TRE GFP DEST	This paper	N/A
MSCV DEST IRES PURO	This paper	N/A
MSCV NTAP DEST IRES PURO	(Behrends et al., 2010)	Addgene plasmid # 41033
MSCV mCherry-ER (KDEL) IRES puro	This paper	N/A
MSCV-SV-tTA	(Liu et al., 2000)	N/A
GST-CCPG1 NTD (bacterial) (1-230)	This paper	N/A
GST-GABARAP mtLDS Y49A L50A	This paper	N/A
MSCV NTAP CCPG1 Human CCPG1 1-757	This paper	N/A
MSCV NTAP EV	(Newman et al., 2017)	N/A
p3xFLAG-CMV10-hFIP200 Human FIP200 1-1594	A gift from Noboru Mizushima, Tokyo medical and dental University, Japan (Hara et al., 2008)	Addgene plasmid # 24300
pBabe-BSD mCherry-CCPG1 Human CCPG1 1-757	This paper	N/A
pdcDNA 6x myc CCPG1 Human CCPG1 1-757	This paper	N/A
pdcDNA 6x myc CCPG1 mtFIR1 S22A D23A I24A E25A Human CCPG1 1-757	This paper	N/A
pdcDNA 6x myc CCPG1 mtFIR2 S104A D105A I106A L109A Human CCPG1 1-757	This paper	N/A
pdcDNA 6x myc CCPG1 mtFIR1+2 S22A D23A I24A E25A S104A D105A I106A L109A Human CCPG1 1-757	This paper	N/A
pdcDNA 6x myc CCPG1 NTD CCPG1 Human CCPG1 1-230	This paper	N/A
pdcDNA 6x myc CCPG1 NTD Human CCPG1 1-230 with internal deletions or truncated from C-terminus, as indicated in main text	This paper	N/A
pdcDNA FLAG-FIP200 Human FIP200 1279-1594	This paper	N/A
pEGFP-C1	Clontech	# 6084-1
pEGFP-CCPG1 CCPG1 Human CCPG1 1-757	This paper	N/A
pEGFP-CCPG1 mtLIR W14A I17A Human CCPG1 1-757	This paper	N/A
pEGFP-CCPG1 mtFIR1+2 S22A D23A I24A E25A S104A D105A I106A L109A Human CCPG1 1-757	This paper	N/A
pEGFP-CCPG1 mtLIR + mtFIR1+2 W14A I17A S22A D23A I24A E25A S104A D105A I106A L109A Human CCPG1 1-757	This paper	N/A
pEGFP-CCPG1 NTD Human CCPG11-230	This paper	N/A
pEGFP-CCPG1 ΔNTD Human CCPG1 231-757	This paper	N/A
pmCherry-ER-3	A gift from Michael Davidson, MagLab, USA	Addgene plasmid # 55041
pMXs-puro GFP-DFCP1	A gift from Noboru Mizushima, Tokyo medical and dental University, Japan (Itakura and Mizushima, 2010)	Addgene plasmid # 38269
pRevTRE EGFP	Clontech	# 6137-1
pRevTRE GFP-CCPG1 Human CCPG1 1-757	This paper	N/A
pRevTRE GFP-CCPG1 mtLIR W14A I17A Human CCPG1 1-757	This paper	N/A
pRevTRE GFP-CCPG1 mtFIR1+2 S22A D23A I24A E25A S104A D105A I106A L109A Human CCPG1 1-757	This paper	N/A
pSpCas9(BB)-2A-Puro (PX45) v2.0	A gift from Feng Zhang, Broad Institute, USA (Ran et al., 2013)	Addgene plasmid # 62988

**Software and Algorithms**		

GraphPad Prism 7	GraphPad Software, Inc.	http://www.graphpad.com/
Imaris 8.1	Bitplane	N/A
NIS-Element Advanced Research software	Nikon Instruments	http://www.micron.ox.ac.uk/software/SIMCheck.php
Fiji	NIH	https://imagej.net/Fiji
MaxQuant (Version 1.5.7.4)	(Cox and Mann, 2008)	http://www.coxdocs.org/doku.php?id=maxquant:start
WebMeV	N/A	http://mev.tm4.org/#/welcome
CompPASS	(Sowa et al., 2009)	http://bioplex.hms.harvard.edu/downloadComppass.php
NiS Elements software	Nikon Instruments	https://www.nikoninstruments.com/en_GB/Products/Software/NIS-Elements-Advanced-Research/NIS-Elements-Viewer
Bioformats plugin	Open Microscopy Environment, Dundee, UK	https://docs.openmicroscopy.org/bio-formats/5.7.0/users/imagej/

### Contact for Reagent and Resource Sharing

Additional information and requests for reagents and protocols should be directed to and will be fulfilled by the Lead Contact, Simon Wilkinson (s.wilkinson@ed.ac.uk).

### Experimental Model and Subject Details

#### Mouse Model

*Ccpg1*^tm1a(EUCOMM)Hmgu^ JM8A3.N1ES embryonic stem cells (C57/BL6N background but with agouti coat colour mutation) were obtained from EUCOMM and embryonically microinjected at the MRC IGMM core transgenic facility. Subsequent chimaeras were then mated with C57BL6/N mice from Charles River Laboratories. This method produces first generation mice on a C57/BL6N background (not a mixed strain background). A founder heterozygote was identified by PCR genotyping. Mice were subsequently maintained on a C57BL6/N background by intercrossing or backcrossing to C57BL6/N. Three generations of backcrosses were performed before the breeding program to generate experimental animals was initiated. The breeding colony was structured by crossing heterozygous mice with heterozygous mice in order to generate wild-type, heterozygous and homozygous offspring. Mice from the same litter (age-matched “littermates”) were considered to have an essentially identical genetic background. Heterozygous offspring were used to regenerate breeding programmes when parents aged more than 6 months. When wild-type and homozygous offspring were identified in a given litter, a pair of such mice was sacrificed contemporaneously by cervical dislocation to permit subsequent generation of tissue histology and/or pancreatic samples for macromolecule extraction, as described elsewhere in this STAR Methods. Each immutable pair of mice was considered n = 1. Pairs were always internally sex-matched (although different pairs in an experiment may be of different sex). Experiments were performed by collecting multiple such pairs of samples, necessarily from different litters and/or different pairs of parental mice.

A sole deviation from the above strategy was amylase analysis where plasma was not from littermate mice and was collected in random groupings, after sacrifice by rising carbon dioxide concentration, on different days.

During the above breeding and all subsequent breeding, bespoke genotyping assays for CCPG1 wild-type and gene trap variants were developed by Transnetyx and all genotyping was performed from ear notching. Mice were bred under standard husbandry conditions in standard cages with environmental enrichment. These mice were created under the authority of UK Home Office Project Licence (MRC IGMM core facility) permitting creation of new strains, after local ethical review. The established line was bred under the authority of a UK Home Office Project Licence held by Simon Wilkinson. The established line was checked on a regular basis for any health concerns associated with the mutant status. No such concerns were raised with mice up to 48 weeks of age.

#### Cell Lines and Culture

All lines were cultured at 37°C in 5 % CO_2_ and with full DMEM supplemented with 10% FBS and penicillin/streptomycin. Amino acid starvations were performed by washing cells three times in Earle’s buffered salt solution (EBSS) before culture in fresh EBSS for the indicated time. Cells were tested every two months to confirm the absence of mycoplasma contamination. A549-EcoR (neoR), HEK293T and HeLa-EcoR (puroR) cells were from laboratory stocks that were verified by microsatellite genotyping. EcoR indicates that the cell line stably expresses the ecotropic receptor to facilitate transduction with ecotropic retrovirus, selected for with the indicated antibiotic resistance marker. All ecotropic virus used was packaged in Phoenix-Eco cells from laboratory stocks. HeLa-TetOff cells were created by infection of HeLa-EcoR cells with MSCV-SV-tTA ecotropic virus and selection in G418. *Atg13* null MEFs and *Ulk1/2* double knockout MEFs were a gift of Noor Gammoh and verified by immunoblotting for deleted proteins as shown in Figure S2A. A549 WT and A549 Δ*ATG5* cell clones were as described in a forthcoming study from this laboratory (Newman et al., 2017). A549 Δ*CCPG1* cells were clonally selected after co-nucleofection of A549 with pSpCas9(BB)-2A-Puro (PX45) v2.0 and gRNA-CCPG1-3, and 24 h selection in 2.5 μg/ml puromycin, as described (Newman et al. Nature Communications 2017, in press). HeLa Δ*CCPG1* - 1 and 2 subclones were generated by clonal selection after Lipofectamine 2000 co-transfection of pSpCas9(BB)-2A-Puro (PX45) v2.0 and gRNA-CCPG1-3 and then 24 h selection in 2.5 μg/ml puromycin. HeLa Δ*ATG5* subclone was generated by clonal selection after Lipofectamine 2000 co-transfection of pSpCas9(BB)-2A-Puro (PX45) v2.0 and gRNA-Atg5-2 and then 24 h selection in 2.5 μg/ml puromycin. A549 NTAP-CCPG1 (FLAG- and HA-dual tagged CCPG1) lines were generated by transduction of A549-EcoR with ecotropic MSCV-NTAP CCPG1 virus and selection in 1.25 μg/ml puromycin. This line was further transduced with ecotropic virus derived from MSCV mCherry-ER3 IRES puro or pMXs-puro GFP-DFCP1, in order to yield A549-NTAP-CCPG1 mCherry-ER (KDEL) and A549-NTAP-CCPG1 GFP-DFCP1 lines, respectively, with no additional selection. HeLa cell lines expressing GFP or GFP-CCPG1, and mutant derivatives of GFP-CCPG1, were generated by transduction of HeLa Tet-Off with ecotropic retrovirus derived from the pREV-TRE-GFP series of plasmids and selection in 200 μg/ml hygromycin. These cells were cultured as stable expressers of GFP or CCPG1, in the absence of doxycycline, and used for experiments within one month of derivation. These cells were further derivatised for 3D-SIM experiment by infection with ecotropic virus derived from MSCV mCherry-ER3 IRES puro, with no additional selection.

### Method Details

#### Chemicals

DTT, Tunicamycin and Bafilomycin A1 were were stored as frozen stock aliquots dissolved in milliQ-water (DTT) or DMSO.

#### Antibodies for Immuno-techniques

Antibody information is found in the accompanying KRT. The application of each antibody can be seen with the following key: IB) immunoblot, IP) immunoprecipitation, IHC) immunohistochemistry, IF) immunofluorescence.

CCPG1 anti-serum was generated by the Eurogentec Double X programme. An N-terminal and internal peptide region were created and used for injections. Final antibody was affinity-purified on N-terminal peptide. The sequences of the two peptides are as shown:

N-terminal peptide seq: H-MSENSSDSDSSC-NH_2_ conjugated to KLH by MBS linker

Internal peptide seq: H-CTEPSKELSKRQFSSG-NH_2_ conjugated to KLH by MBS linker

#### Plasmid Generation

The majority of cloning was performed by using the Gateway method as per standard protocols (https://www.thermofisher.com/uk/en/home/life-science/cloning/gateway-cloning/protocols.html). Detailed sequence maps are available from authors upon request.

#### RNA Interference

10^5^ A549-EcoR were seeded overnight in 35mm diameter wells. Cells were transfected for 8 h with Oligofectamine and 50 pmoles of siRNA, according to the manufacturer’s instructions.

#### Protein-Protein Interaction Mass Spectrometry

Four 15 cm cell culture dishes of A549-NTAP-CCPG1 cells were washed and harvested with ice-cold PBS followed by storage at -80 °C and then lysed in 4 ml mass spectrometry lysis buffer (MSLB; 50 mM Tris-HCl pH 7.5, 150 mM NaCl, 0.5% Nonidet P40, EDTA-free complete protease inhibitor cocktail). Lysates were cleared by centrifugation and 0.45 μm spin filtration. Anti-HA-agarose (60 μL slurry) was used for immunoprecipitation overnight at 4°C on a rotating wheel. Samples were washed five times with 1 ml MSLB followed by five washes with PBS and elution with 150 μL HA peptide (250 μg/ml). Eluates were processed and analysed as previously described (Sowa et al., 2009, Behrends et al., 2010). In brief, precipitation of protein with trichloroacetic acid preceded trypsin digestion and desalting by stage tips. Samples were analysed in technical duplicates on a LTQ Velos (Thermo Scientific). Spectra were identified by Sequest searches followed by target-decoy filtering and linear discriminant analysis as previously described (Huttlin et al., 2010). Peptides that could be assigned to more than one protein in the database were assembled into proteins according to parsimony principles. For CompPASS analysis, we employed 33 unrelated bait proteins that were all processed in the same way in A549 cells. Weighted and normalized D-scores (WDN-score) were calculated based on average peptide spectral matches (APSMs).

#### CCPG1 Peptide Array

Cellulose membrane spotted with 15-mer peptides was obtained from JPT Peptide Technologies GmbH (for full list of sequences see Figure S1B). Membrane was blocked with 5 % BSA/TBST and probed with recombinant FIP200 for 1 hour. Membrane was washed 3 x 5 minutes in TBST, then probed with rabbit anti-FIP200 for 1 hour at room temperature in 2% BSA/TBST, washed again and then similarly probed with HRP-linked anti-rabbit secondary antibody. Washed membranes were then developed with ECL and exposed to X-ray film.

#### GST Fusion Protein Production and Affinity Precipitation Assays

Rosetta II (DE3) cells were transformed with bacterial GST-fusion expression vectors. Cultures grown in L-broth were induced with 1 mM IPTG for two hours. Cells were suspended in 50 mM Tris-HCl, pH 7.5, 150 mM NaCl, 1 mM EDTA, and 0.1 mM PMSF and Complete Protease Inhibitor. Cells were sonicated and then IGEPAL detergent was spiked in to a final concentration of 1 % and incubated for 1 h at 4°C to effect complete lysis. Insoluble material was removed by centrifugation at 17 000 g for 20 min at 4°C. Glutathione Sepharose 4B beads were washed 3 times in IGEPAL IP buffer (50mM Tris-HCl, pH 7.5, 150 mM NaCl, 0.5% IGEPAL, 2 mM activated sodium orthovanadate, 20mM NaF, 10 mM sodium pyrophosphate and Complete Protease Inhibitor), using 20 μl per reaction. GST-fusion protein containing lysate was added to reaction, incubated for 1 h at 4°C and then washed three times in IGEPAL buffer to batch pre-purify the immobilised GST-fusion protein on the beads. Recombinant protein or HEK lysate prepared in IGEPAL IP buffer was then added to aliquots of beads and reaction volume topped up to 1 ml with IGEPAL IP buffer. Reactions were incubated with rotation at 4°C for 2-18 h, then washed 3 times with IGEPAL IP buffer before boiling beads in Laemmli buffer for analysis via immunoblotting.

#### Co-immunoprecipitation Assays

HEK293T or HeLa cells were transfected with 1 μg of a single protein expressing or empty vector plasmid DNA, per 6-well, with Lipofectamine 2000, using manufacturer’s recommendations, for 24 h. Cells were lysed using IGEPAL IP buffer and then centrifuged at 17 000 g for 15 min at 4°C. Equal amounts of required supernatants containing the proteins to test interaction between were added together, mixed and 0.05 volumes removed for use as input controls. Mouse FLAG-M2 agarose beads, rabbit-myc agarose beads or GFP-Trap_MA beads were washed 3 times in IGEPAL IP buffer and 20 μl added per binding reaction. Reactions were incubated with rotation at 4°C for 2 h (or 15 h for myc beads), then washed 3 times with IGEPAL IP buffer by centrifugation at 5500g (FLAG/myc) or magnetic separation (GFP) before boiling immunoprecipitates from beads into Laemmli buffer for analysis via immunoblotting.

#### Immunoblotting

For direct immunoblotting analysis, cells were either lysed in RIPA buffer (50mM Tris-HCl, pH 7.5, 150 mM NaCl, 1 % IGEPAL and complete protease inhibitors) or SDS buffer (4 % SDS, 150 mM NaCl, 50 mM Tris, pH 7.5). All samples were diluted with Laemmli buffer to 1 x before heating at 95°C for 3 min before gel electrophoresis. Samples were separated using SDS-PAGE employing MOPS NuPAGE 4-12% gels from Invitrogen as directed by the manufacturer. An exception to this was endogenous CCPG1 blotting, which was in some instances performed using 3-8% Tris-Acetate NuPAGE gels from Invitrogen. This provides superior resolution of endogenous CCPG1 from background bands with the antiserum used. Gels were transferred by wet blotting onto Protran nitrocellulose membrane. Membranes were blocked with 5 % w/v non-fat dry milk or 5 % w/v bovine serum albumin in TBST and generally probed overnight with primary antibody at 1:2000 in 2 % w/v of the same blocking agent in TBST + 0.05% sodium azide. Membranes were washed 3 x 15 min with TBST and incubated for 1 hour at room temperature with cognate secondary HRP-linked antibody at 1:4000. Blots were then washed again and developed using X-ray film with either standard ECL or, for low signals, ECL prime.

#### Immunohistochemistry

Freshly excised pancreata were fixed overnight at room temperature by immersion in 10% neutral buffered formalin. Samples were then embedded in paraffin and FFPE (formalin-fixed paraffin-embedded) sections cut for standard haematoxylin and eosin staining or for immunohistochemistry. Samples were stained using DAB on the Leica Bond Max automated immunostainer platform, according to manufacturer’s instructions and using standard rabbit antibody detection reagents (Leica Bond Polymer Refine Detection). Leica pH 6.0 citrate buffer was used for epitope retrieval. The standard protocol settings used were: wash 10 min, peroxide block 5 min, primary antibody 60 min, polymer 15 min, mixed DAB 10 min, hematoxylin 5 min. Ki67 antibody was used at 1:250. PDI primary antibody was used at 1:100. Ki67 staining was quantified by single blinded assessment of the number of DAB positive nuclei in at least 1000 acinar cells per animal.

#### RNA Isolation

RNA isolation from cell lines was performed using the Qiagen RNeasy kit and QIAshredder columns for homogenisation, all according to manufacturer’s instructions. RNA isolation from pancreas was performed by snap freezing freshly excised pancreata in liquid nitrogen. These were wrapped in tinfoil and bathed briefly in liquid nitrogen and then pulverised with a precooled metal block before immersing pulverised contents in liquid nitrogen in a cold-resistant mortar. A precooled pestle was then used to grind samples for 3 x 5 seconds under constant liquid nitrogen. Powdered pancreata were then scraped into a tube of 1 ml Trizol per 50 mg initial wet weight of tissue. Trizol samples were spun in a microfuge at 12 000g for 10 min at 4°C. The supernatant was retained and 200 μl chloroform added per 1 ml. This was vortexed slowly until well mixed and left to rest for 2 min at room temperature. This sample was then centrifuged at 12 000g for 15 min at 4°C. The subsequent aqueous phase was retained. 0.5 ml isopropanol was added per original 1 ml of Trizol and the sample gently inverted/mixed 10 times. The sample was rested for 10 min at room temperature and then microcentrifuged at 10 000g for 10 min at 4°C. The pellet was washed with 500 μl of 75% ethanol and spun again. The pellet was air dryed for 10 min, then resuspended in 100 μl of RNAse-free water. 250 μl of RLT buffer from the Qiagen RNeasy kit was then added to the sample and a final clean up performed by following the Qiagen RNeasy kit manufacturer’s instructions. Integrity of pancreatic RNA was assessed by agarose gel electrophoresis.

#### Whole Pancreatic Protein Isolation

Fresh excised pancreata were snap frozen in liquid nitrogen, wrapped in tinfoil and flattened with a precooled metal block then ground under liquid nitrogen using a cold-resistant mortar and pestle for 3 x 5 minutes. Powdered pancreata were scraped from pestle into a room-temperature tube of SDS lysis buffer (4% SDS, 150mM NaCl, 50mM Tris pH 7.5, 200 μl per 50 mg initial wet weight of tissue). Samples were vortexed, homogenised with a 21G needle, boiled for 5 minutes and then sonicated before spinning in a microcentrifuge at 17 000 g for 15 min at room temperature. Any visible fat contamination was removed from the surface of the supernatant, the supernatant was retained as the soluble fraction. The pellet was washed with another volume of lysis buffer and spun at 17 000 g for 10 min. The washed pellet was resuspended in a volume equivalent to the supernatant of urea buffer (8M Urea, 1% SDS, Tris-HCl pH 8.0, 10mM DTT) and boiled with occasional vortexing until the solution was wholly clarified in all samples (time generally determined by the *Ccpg1* genetrap homozygote samples in each pair of wild-type and mutant pancreas samples). These samples were either immunoblotted or subjected to label free quantification mass spectrometry.

#### Pancreatic Protein Mass Spectrometry

Both soluble and insoluble samples were cleared of detergents, reduced, alkylated and digested according to the filter-aided-sample-processing protocol as previously reported (Wisniewski and Rakus, 2014). In brief, the proteins were reduced, alkylated, and digested with GluC. The resulting peptide samples were separated on a Nanoflow Ultimate 3000 LC (Thermo) coupled online to a Q-Exactive plus mass spectrometer (Thermo). The self-packed HPLC C18-reversed phase column used was 20 cm long, 75 μm inner diameter, packed with a UChrom C18 1.8μm resin. The peptide mixtures were loaded at 400nL/min onto the column. The peptides were eluted at a constant flow rate of 250nL/min over a period of 120 min with a multi-segment linear gradient of 2-40% buffer B (80% Acetonitrile and 0.05% acetic acid) in positive ion mode. A data-dependent automatic “top 12” method was employed with a survey scan (MS) in the mass range of a mass-to-charge ratio (m/z) of 350-1600 which selected the twelve most intense ions for collision induced fragmentation and acquisition of tandem mass spectra. The raw mass spectrometric data files from LC-MS/MS were analysed using MaxQuant (Version 1.5.7.4). GluC was selected as enzyme, first search and fragmentation spectra were searched with 20 ppm mass accuracy, recalibrated *in silico* and subsequently searched with the parent ion standard mass accuracy reduced to less than 1 ppm. Fixed modifications were carbamylation of cysteines, variable modifications were methionine oxidation and protein N-terminal acetylation. We allowed for two missed cleavages. The data were searched against a murine database (Uniprot version 2016_11), a reversed database and a contaminant data base (152 entries). A false discovery rate (FDR) of 0.01 at the peptide and protein level. FDR was estimated by searching a reversed and forward database. The LFQ values were determined by the MaxQuant software suite. For data analysis of the insoluble protein fractions, at least one sample must have identified a protein species with >2 unique peptides. Otherwise, that protein identification was removed from the dataset. Identified contaminants were also removed. The LFQ values were normalised according to the measure protein concentration of the corresponding soluble sample in SDS lysis buffer. Then the top 20 proteins clustered by MAD analysis on basis of LFQ values using WebMeV were presented in the heatmap in Figure 6.

#### qRT-PCR

1st strand cDNA was synthesised from total RNA using qScript cDNA SuperMix according to manufacturer’s instructions. qPCR was performed on an Applied Biosystems StepOne Plus Real-Time PCR System using SYBR green detection via the DyNAmo HS SYBR Green qPCR Kit according to manufacturer’s instructions. Standard cycling parameters were 40 cycles of 95°C for 15 seconds, 60°C for 1 min. ΔΔCt method was used to calculate relative abundances of specific transcripts between samples, normalised to 18S rRNA. For list of primers see Table S3.

#### Mouse Plasma Amylase Analysis

Blood samples were taken by cardiac puncture of mice which had been culled through rising CO_2_ concentration. Blood was centrifuged at 1400 g for 10 min at 4°C in a benchtop microncentriufuge in EDTA-coated tubes. Supernatant (plasma) was frozen and subsequently analysed for amylase. Plasma α-Amylase was determined by a commercial kit (Alpha Laboratories Ltd., Eastleigh, UK) adapted for use on a Cobas Fara centrifugal analyser (Roche Diagnostics Ltd, Welwyn Garden City, UK.). The method utilises 2-chloro-pnitrophenyl-α-D-maltotrioside as the substrate. α-Amylase hydrolyzes the 2-chloro-p-nitrophenyl-α-D-maltotrioside to release 2-chloro-p-nitrophenol and produce 2-chloro-p-nitrophenyl-α-D-maltoside, maltotriose, and glucose. The rate of formation of the 2-chloro-p-nitrophenol can be detected spectrophotometrically at 405 nm to give a direct measurement of α-amylase activity in the sample. Within run precision was CV < 4% while intra-batch precision was CV < 5%.

#### Immunofluorescence

Cells were grown on 16mm glass coverslips unless otherwise specified. Cells were fixed by washing and then incubating with 4% paraformaldehyde in PBS, pH 7.2 at room temperature for 10 min. Cells were permeabilised with either methanol, kept at -20°C for 5 minutes (LC3B and LAMP2) or with 0.25% Triton X-100 in PBS for 15 minutes at room temperature (all other antibodies). Cells were then washed twice with PBS and incubated for 1 h at 37°C in 1% BSA/PBS/0.02% sodium azide containing primary antibody (all at a 1/200 dilution except for WIPI2 and LAMP2 antibodies, which were both used at 1/400). Cells were then washed for 3 x 5 min in PBS with gentle agitation before incubation with secondary antibody (goat IgG H+L AlexaFluor 488, 594 or 647) at 1/400 dilution in 1% BSA/PBS/0.02% sodium azide for 1 hour at room temperature. Cells were then washed for 3 x 5 min in PBS with gentle agitation and then mounted in Dako fluorescent mounting medium (Dako) onto glass slides. Where stated, DAPI was added at 1/5000 dilution during the penultimate wash.

#### Loading of ER-Tracker Red

ER-tracker Red was prepared and stored as recommended by the manufacturer’s manual. Cells were seeded on 16mm glass coverslips and incubated with ER-tracker to a final concentration of 1 μM for the final 30 min of treatment, at 37°C in 5 % CO_2_. Cells were washed once with PBS prior to paraformaldehyde fixation, then washed again and mounted in Dako fluorescent mounting medium onto glass slides.

#### CARS Microscopy

Freshly excised pancreas was washed with PBS and then diced with a scalpel blade in 10% neutral buffered formalin for 1 hour. Pancreas chunks were then washed extensively in PBS and mounted on a microscope slide and analysed by CARS microscopy. Briefly, a picoEmerald (APE) laser provided both a tunable pump laser (720–990 nm, 7 ps, 80 MHz repetition rate) and a spatially overlapped second beam termed the Stokes laser (1064, nm, 5–6 ps and 80 MHz repetition rate). The laser was inserted into an Olympus FV1000 microscope coupled to an Olympus XLPL25XWMP N.A. 1.05 objective lens using a short-pass 690 nm dichroic mirror (Olympus KeyMed, UK). Back scattered CARS signals were filtered using the following series of filters: FF552-Di02 (Semrock, NY), t640lpxr and ET687/95m (Chroma Technology Corp., VT). The pump laser was tuned to 816.8 or 812.2 nm and used 50 mW power as measured at the objective, whilst the Stokes laser used 20 mW power as measured at the objective. Images were recorded using the FV10-ASW software (Olympus KeyMed, UK).

#### Confocal Microscopy

Images were captured with a Nikon A1R TiE confocal microscope using either a 60x 1.4 NÅ (ER-tracker staining) or 100x 1.4 NÅ (co-localisation images) objective (Nikon Instruments, UK). All confocal images are shown as z-projections of at least 3 z-steps.

#### 3D-Structured Illumination Microscopy (3D-SIM)

Samples were prepared on high precision cover-glass. Cells were fixed, permeabilised and stained as described above. Cells were incubated with goat anti-rabbit IgG H+L AlexaFluor 647 at 1/400 dilution in 1% BSA/PBS/0.02% sodium azide for 1 hour at room temperature. Coverslips were mounted in Fluoroshield mounting medium. 3D SIM images were acquired on an N-SIM (Nikon Instruments, UK) using a 100x 1.49NA lens and refractive index matched immersion oil (Nikon Instruments, UK). Images were captured using an Andor DU-897X-5254 camera using 488, 561 and 640nm laser lines (Andor Technologies, UK). Z-step size for Z stacks was set to 0.120 um as required by manufacturer’s software. For each focal plane, 15 images (5 phases, 3 angles) were captured with the NIS-Elements software. SIM image processing, reconstruction and analysis were carried out using the N-SIM module of the NIS-Element Advanced Research software. Images were reconstructed using NiS Elements software from a z stack comprising of no less than 1 μm of optical sections. In all SIM image reconstructions the Wiener and Apodization filter parameters were kept constant.

#### Time-Lapse Fluorescence Microscopy

Cells were seeded onto 35mm glass bottom dishes and imaged in a humidified stage top environmental chamber (Oko-lab, IT) at 37°C, 5% CO_2_. Stable cell lines with GFP-CCPG1 were loaded with ER-tracker Red 30 minutes prior to the first acquisition. High speed confocal imaging was carried out using a Dragonfly spinning disk confocal microscope (Andor Technologies, UK) mounted on a Nikon Ti-E Stand using a 100x 1.4 NÅ objective (Nikon Instruments, UK). Focus stability was maintained during imaging using the Perfect Focus system. Cells were imaged using a 488 nm and 561 nm laser line (both at 250 ms exposure and 2.5% transmission). Images were collected onto an iXon888 EMCCD camera (Andor Technologies, UK) at a frame rate of 20 seconds. Images were collected over a 0.5 μm Volume were sampled at Nyquist, with the Z step size set to 0.2 μm. Data was saved in the native.ims format, 3D data analysis was carried out in Imaris 8.1 (Bitplane, Switzerland). Data was imported into Fiji using the Bioformats plugin.

#### Transmission Electron Microscopy

Freshly excised pancreata were washed briefly in PBS and then diced into 1 mm^3^ chunks in 3% EM-grade gluteraldehyde, 0.2M sucrose, 0.1M sodium cacodylate pH 7.4. These were left in fixative overnight and then changed to fresh 0.1M cacodylate buffer. Tissue was then embedded in Epon resin, ultrathin sections cut and osmicated. Sections were then analysed on a Philips / FEI CM120 Biotwin transmission electron microscope.

### Quantifications and Statistical Analyses

#### In Vitro

##### Imaging Quantification Parameters (General)

For cell lines, quantification was performed as described in figure legends or in the relevant sections below. Quantifications were performed on z-stack projections. All quantifications were performed on a minimum of 80 cells across three biological replicates and the standard error of the mean was determined for each data set. Cells were single blind scored.

##### ER-Tracker Red Quantification

For quantification of ER-tracker Red staining, images were manually background subtracted and a threshold applied through Fiji as described previously (Khaminets et al., 2015). Cells were selected by drawing around the boundary of GFP-CCPG1 expressing cells (for over-expression experiments) or the boundary of randomly selected cells as seen in bright field images (CRISPR lines) and only the area inside the boundary was quantified. The percentage of ER signal above threshold was normalised to control cell lines and plotted as a ratio.

##### Quantitation of GFP-CCPG1 Puncta and Co-localisation Analyses

Quantification of puncta was performed using the Spots Detection function of Imaris software (Bitplane/Andor). Co-localisation analysis for the specifically punctate signal of GFP-CCPG1 with endogenous LC3B signal was performed by using the Spots Detection followed by Co-loc functions of the Imaris XT software (Bitplane/Andor). The measurements were made on randomly selected fields of view. The colocalisation parameter is a Pearson’s coefficient calculated on a per cell basis.

##### Statistical Analysis

All *in vitro* experiments were performed as independent biological replicates and the n value given in appropriate figure legends. Further details on the statistical treatment of imaging based assays, including minimum number of cells or features counted are given in the STAR Methods section above. All statistical analysis was performed with GraphPad Prism 7 and graphs generated through Microsoft Excel. Testing was performed based upon an assumed pair of samples with similar variances was used to address the significance of differences between samples, either one sample t-testing versus a hypothetical normalised mean of 1 for experiments where the control value was set to 1 for normalisation purposes in each independent replicate, or standard Student’s t-test in other instances. Tests were always two-tailed. Where multiple samples were compared amongst each other, an unpaired one-way ANOVA was performed followed by the application of Tukey’s post-hoc test. If more than one independent variable was changed within the experiment, then a two-way ANOVA was performed followed by the application of Tukey’s post-hoc test. p-values < 0.05 were considered to be significant.

#### In Vivo

Where representative histology rather than quantification is given in the main figures, this is representative of minimally three such pairs of mice. The qualitative differences in macroscopic appearance of the pancreas were noted and found to be completely consistent across all pairs of mice sacrificed during this project for any purpose.

Quantitative *in vivo* experiments were analysed the same way as the above *in vitro* experiments, via t-testing, with the same underlying assumptions of data variance made. The two groupings for *in vivo* comparisons each contained the same number of mice. In the majority of instances, these groupings are built with individual pairs of mice from the same litter (i.e. wild-type and gene trap homozygous comparators, generated from the mating of heterozygous parents). Each pair of mice was considered an independent experimental comparison for statistical purposes. Figure legends indicate the number of such pairs, n, and use the phrase “littermates” to denote this selection method. The assessor was always blinded to sample identity when analysing pathology or performing molecular analyses.

## Supplemental Information

Document S1. Figures S1–S7

Table S1. LC-MS/MS Summary Data from Analysis of HA-CCPG1 Immunoprecipitates, Related to Figure 2 Lysates from A549 NTAP (FLAG-HA)-CCPG1 cells were immunoprecipitated using anti-HA antibody and immunoprecipitates subjected to LC-MS/MS and CompPASS analysis. Interactors are sorted by highest WDN score to lowest. Interactors highlighted in green are gated “hits” based upon the criteria of a WDN score above 0.8, a *Z* score above 1, and detection of more than one peptide in at least one of two technical replicates. Common keratin contaminants were excluded.

Table S2. Label-Free Quantification LC-MS/MS Data of Insoluble Fraction of Mouse Pancreas, Related to Figure 6 Label-free quantification (LFQ) LC-MS/MS was performed to analyze the material enriched in the insoluble fraction, using littermate-paired and sex-matched pairs of wild-type and CCPG1-deficient mice (n = 3). LFQ counts are presented for each mouse. Pairs of mice are denoted 1, 2, and 3.

Table S3. qRT-PCR Primers and siRNA Oligonucleotides, Related to the STAR Methods Source and nucleic acid sequence of DNA oligonucleotides used in qRT-PCR analyses, and the source and targeting sequences of double-stranded RNA oligonucleotides used in RNAi experiments.

Movie S1. Live Imaging of CCPG1 Foci on the ER, Related to Figure 4 HeLa GFP-CCPG1 cells were loaded with ER tracker red dye, starved in EBSS, and imaged for both fluorophores by time-lapse spinning disk confocal microscopy. z stacked images composed of three individual sections were obtained every 20 s. Three representative movies are shown sequentially. Start times of each movie are shown by the timer in the top left corner.

Document S2. Article plus Supplemental Information
