# Supplementary material for: CCPG1 Is a Non-canonical Autophagy Cargo Receptor Essential for ER-Phagy and Pancreatic ER Proteostasis
Source: Dev Cell. 2018 Jan 22;44(2):217–232.e11. doi: 10.1016/j.devcel.2017.11.024 (PMC5791736; doi:10.1016/j.devcel.2017.11.024)
Supplement: Document S1. Figures S1–S7 [file mmc1.pdf]

Developmental Cell, Volume *44*

Supplemental Information

CCPG1 Is a Non-canonical Autophagy Cargo Receptor Essential for ER-Phagy and Pancreatic ER Proteostasis

Matthew D. Smith, Margaret E. Harley, Alain J. Kemp, Jimi Wills, Martin Lee, Mark Arends, Alex von Kriegsheim, Christian Behrends, and Simon Wilkinson

Supplemental Figure S1, related to Figures 2 and 3

Further analysis of FIP200-CCPG1 binding

A) WT, Δ*ATG5* or Δ*CCPG1* clones of A549 cells were subjected to endogenous immunoprecipitation for CCPG1 and then immunoblotted for CCPG1 and FIP200 (IgG = negative control IgG). FIP200 only immunoprecipitates if CCPG1 is present. This assay validates the CCPG1 antiserum used throughout this study for immunoblotting and endogenous immunoprecipitation (n.s. = non-specific band).

B) Full list of peptides constituting the array analysed in main Figure 3A with a reproduction of the array from this Figure for cross-reference purposes.

C) Human CCPG1 FIR2 motif fits the consensus established for yeast Atg11BR motifs. Of known yeast Atg11BR motifs, it aligns most closely to that from Atg34 (shown).

A

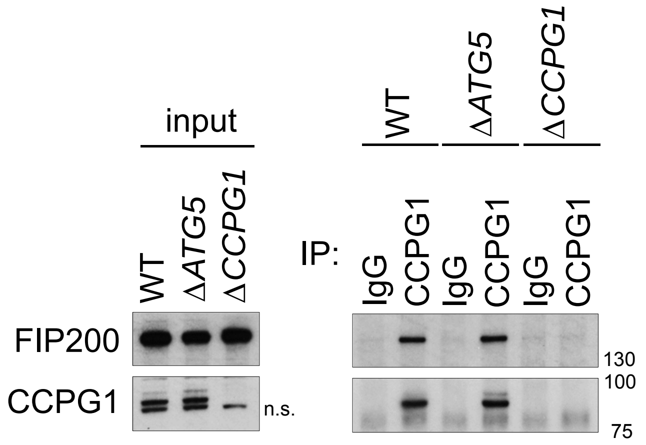

B

1	MSENSSDSDSSCGWT	15	QGESSQNGTVLMEET	29	KLEEIGNQEVVIVEE	43	NQPSPAFRRRRARKK
2	SSDSDSSCGWTVISH	16	SQNGTVLMEETAYPA	30	IGNQEVVIVEEAQSS	44	PAFRRRRARKKTVSA
3	DSSCGWTVISHEGSD	17	TVLMEETAYPALEET	31	EVVIVEEAQSSEDFN	45	RRRARKKTVSASESE
4	GWTVISHEGSDIEML	18	EETAYPALEETSSTI	32	VEEAQSSEDFNMGSS	46	RKKTVSASESEDRLV
5	ISHEGSDIEMLNSVT	19	YPALEETSSTIEAEE	33	QSSEDFNMGSSSSSQ	47	VSASESEDRLVAEQE
6	GSDIEMLNSVTPTDS	20	EETSSTIEAEEQKIP	34	DFNMGSSSSSQYTFC	48	ESEDRLVAEQETEPS
7	EMLNSVTPTDSCEPA	21	STIEAEEQKIPEDSI	35	GSSSSSQYTFCQPET	49	RLVAEQETEPSKELS
8	SVTPTDSCEPAPECS	22	AEEQKIPEDSIYIGT	36	SSQYTFCQPETVFSS	50	EQETEPSKELSKRQF
9	TDSCEPAPECSSLEQ	23	KIPEDSIYIGTASDD	37	TFCQPETVFSSQPSD	51	EPSKELSKRQFSSGL
10	EPAPECSSLEQEELQ	24	DSIYIGTASDDSDIV	38	PETVFSSQPSDDESS	52	ELSKRQFSSGLNKCV
11	ECSSLEQEELQALQI	25	IGTASDDSDIVTLEP	39	FSSQPSDDESSSDET	53	RQFSSGLNKCVILAL
12	LEQEELQALQIEQGE	26	SDDSDIVTLEPPKLE	40	PSDDESSSDETSNQP	54	SGLNKCVILALVIAI
13	ELQALQIEQGESSQN	27	DIVTLEPPKLEEIGN	41	ESSSDETSNQPSPAF	55	KCVILALVIAISMGF
14	LQIEQGESSQNGTVL	28	LEPPKLEEIGNQEVV	42	DETSNQPSPAFRRRR		

15-mer array: CCPG1 (1-231)

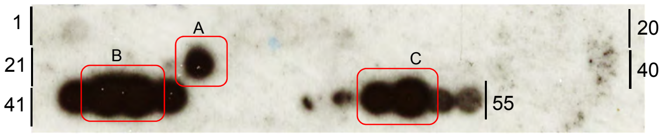

Probe: recombinant FIP200

	101 ... 115
A] peptide 26	**SDDSDIVTLEPPKLE**
	169 ... 187
B] peptides 43-44	NQPS**PAFRRRRARKK**TVSA
	205 ... 223
C] peptides 52-53	ELSK**RQFSSGLNKCV**ILAL

C

– – – – Φ Φ – –

Sc Atg34	DESSIMSTP	Atg11BR
Hs CCPG1	DDSDIVTLE	FIR2

Φ : hydrophobic
- : D/E/S/T

Supplemental Figure S2, related to Figure 3

Further controls for FIP200-CCPG1 binding

A) *Ulk1/2* double knockout (DKO) or *Atg13* knockout (KO) MEFs were transfected with FLAG-FIP200 using Lipofectamine 2000, as previously described for HeLa and HEK293T cells in STAR Methods. Separate populations of each line were transfected with pEGFP-C1 or pEGFP-CCPG1 full length (FL) or point mutations thereof. FLAG-FIP200 lysate was added to each GFP lysate in equal measure and incubated for 2 hours with GFP-Trap beads, whereupon co-precipitation of FLAG-FIP200 was attempted. In both the absence of ULK1/2 or ATG13 protein, CCPG1 was able to bring down FIP200, regardless of whether the LIR motif was ablated or not. In both instances, dependency upon the FIR1 and FIR2 motifs is evident, however (n.s. = non-specific band).

B) HEK293 cells were transfected with GFP or indicated GFP-CCPG1 mutants. GST-GABARAP affinity precipitations were performed from cellular lysates and samples were immunoblotted as shown.

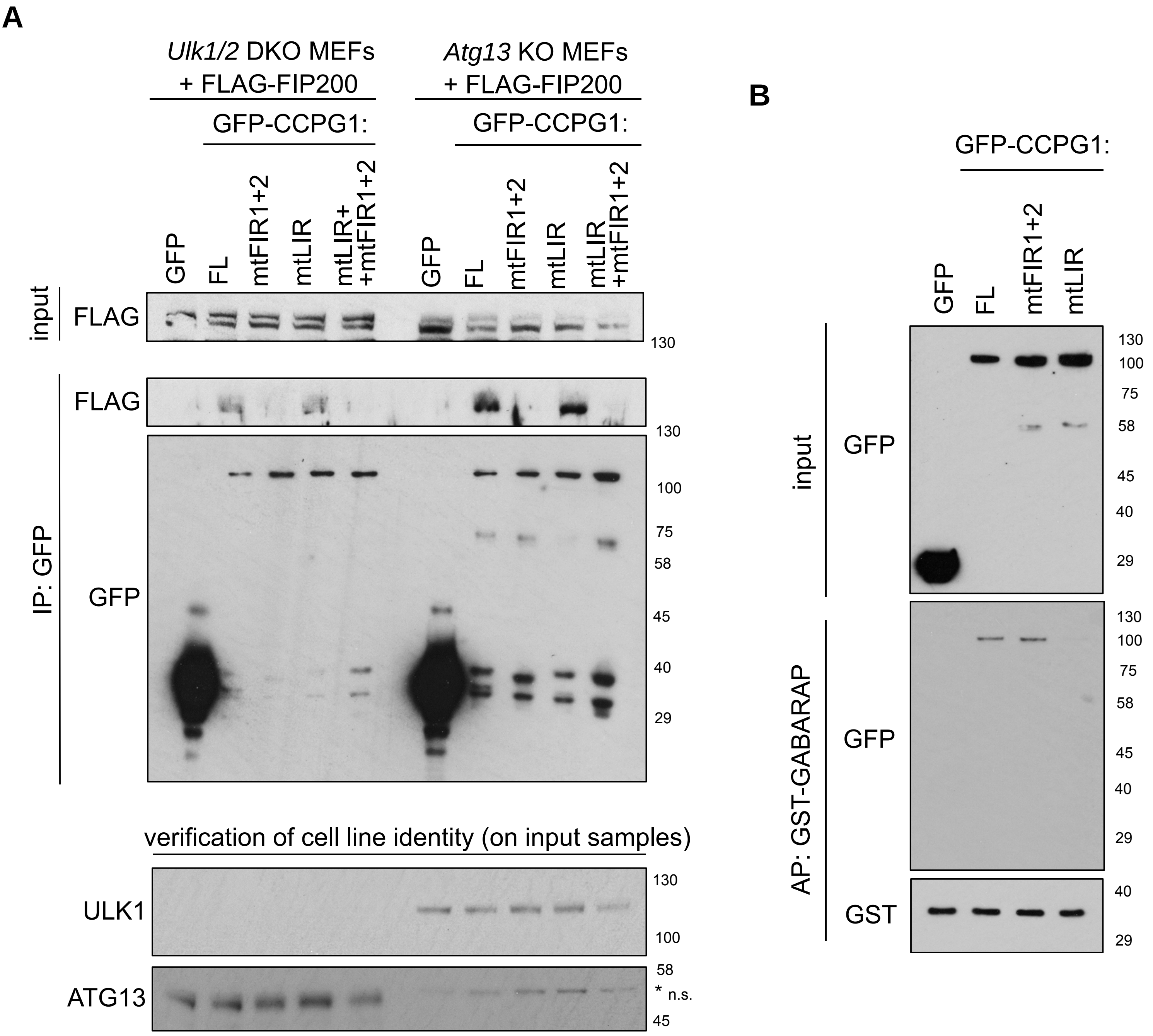
A
Ulk1/2 DKO MEFs
+ FLAG-FIP200
GFP-CCPG1:
Atg13 KO MEFs
+ FLAG-FIP200
GFP-CCPG1:
GFP
FL
mtFIR1+2
mtLIR
mtLIR+
+mtFIR1+2
input
FLAG
130
IP: GFP
FLAG
130
GFP
100
75
58
45
40
29
verification of cell line identity (on input samples)
ULK1
130
100
ATG13
58
* n.s.
45
B
GFP-CCPG1:
GFP
FL
mtFIR1+2
mtLIR
input
GFP
130
100
75
58
45
40
29
AP: GST-GABARAP
GFP
130
100
75
58
45
40
29
GST
40
29

Supplemental Figure S3, related to Figure 4

Further localisation data for CCPG1

A) A549 mCherry-ER cells were starved in EBSS for 1 h and stained for endogenous CCPG1, and then imaged by confocal microscopy. Scale Bar = 10 µm.

B) A549 NTAP-CCPG1 cells were left untreated or starved for 1 h in EBSS, then stained for HA.

C) A549 NTAP-CCPG1 or A549 NTAP-CCPG1 GFP-DFCP1 cells were starved for 1 h in EBSS and co-stained for HA and, if indicated, an additional ATG marker, then imaged by confocal microscopy. Scale Bar = 20 µm.

D) A549 NTAP-CCPG1 or A549 NTAP-CCPG1 GFP-DFCP1 cells were starved for 1 h in EBSS and co-stained for HA and, if indicated, an additional one or two staging markers for the autophagy pathway, then imaged by confocal microscopy. Scale Bar = 10 µm.

E) A549 cells were starved for 1 h in EBSS and stained for endogenous CCPG1 and STX17. Scale Bar = 10 µm.

F) A549 mCherry-CCPG1 stable line was starved in EBSS for 1 h and fixed and stained for LAMP2 (lysosomal marker). Scale Bar = 20 µm.

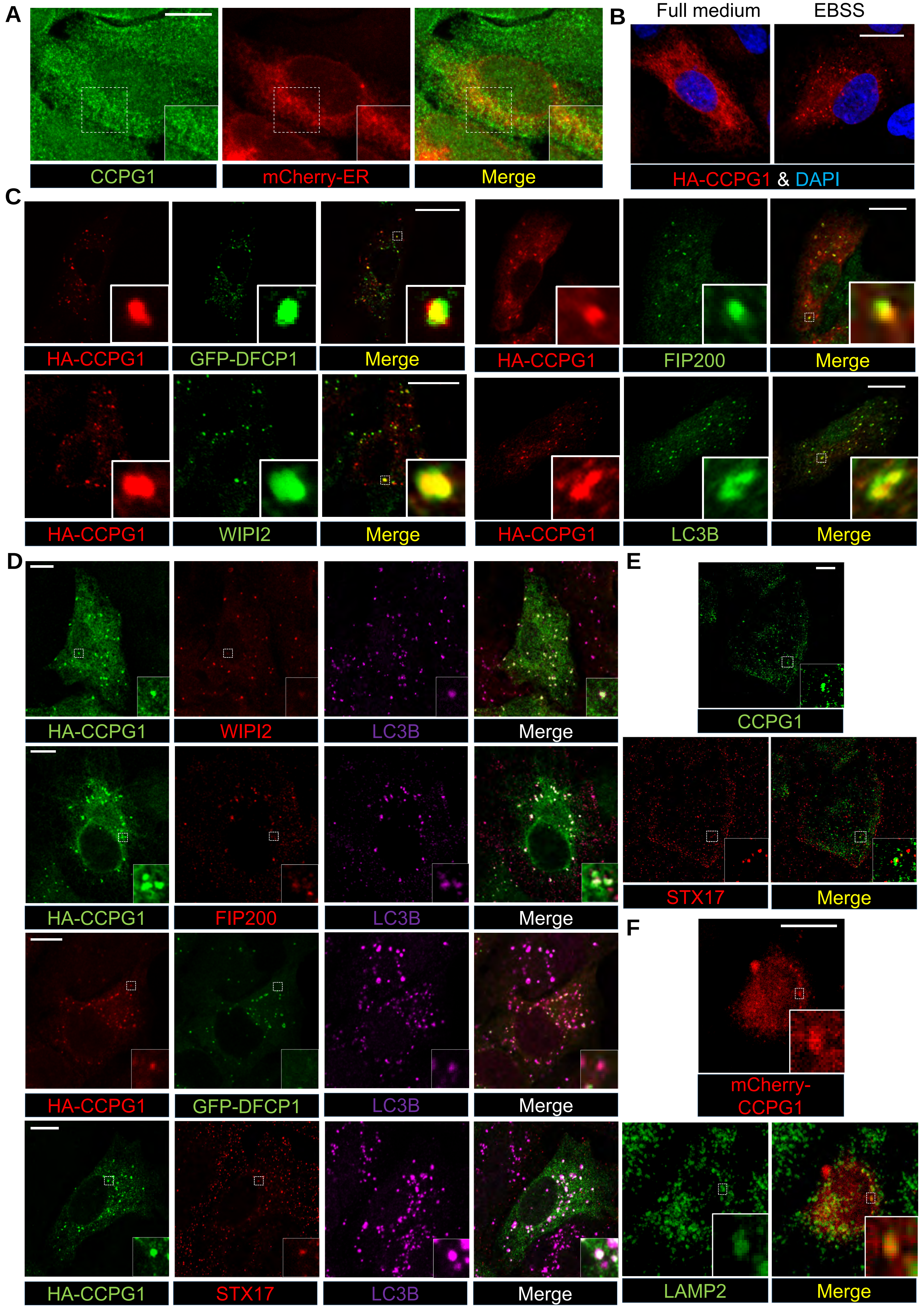
A
CCPG1
mCherry-ER
Merge
B
Full medium
EBSS
HA-CCPG1 & DAPI
C
HA-CCPG1
GFP-DFCP1
Merge
HA-CCPG1
FIP200
Merge
HA-CCPG1
WIPI2
Merge
HA-CCPG1
LC3B
Merge
D
HA-CCPG1
WIPI2
LC3B
Merge
HA-CCPG1
FIP200
LC3B
Merge
HA-CCPG1
GFP-DFCP1
LC3B
Merge
HA-CCPG1
STX17
LC3B
Merge
E
CCPG1
STX17
Merge
F
mCherry-CCPG1
LAMP2
Merge

Supplemental Figure S4, related to Figure 5

Role of ATG5 in CCPG1-driven reduction in ER abundance

A-C) HeLa GFP or GFP-CCPG1 cells were transfected with the indicated siRNA (siCtrl = non-targeting control). A) Representative immunoblot 24 h after transfection of GFP-CCPG1 cells showing knockdown of ATG5 protein. B) Transfected cells were analysed for peripheral ER morphology as in main Figure 5C, quantified in C) (n = 3, ± S.E.M., * $p < 0.05$, # non-significant, one-way ANOVA with Tukey's *post-hoc* test).

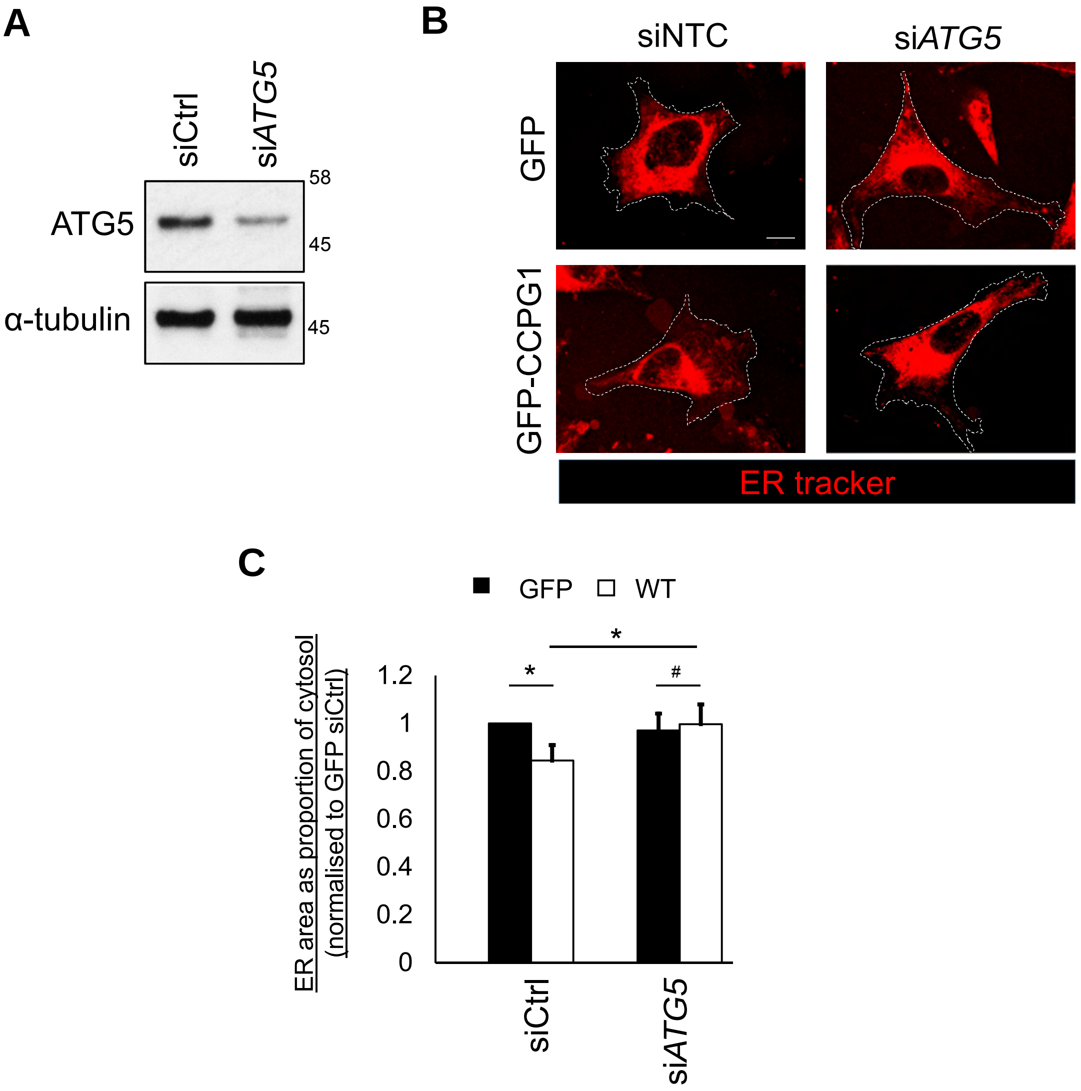

A
siCtrl
siATG5
58
ATG5
45
α-tubulin
45
B
siNTC
siATG5
GFP
GFP-CCPG1
ER tracker
C
GFP
WT
*
*
#
1.2
1
0.8
0.6
0.4
0.2
0
ER area as proportion of cytosol
(normalised to GFP siCtrl)
siCtrl
siATG5

Supplemental Figure S5, related to Figures 6 and 7

Characterisation of new *Ccpg1* hypomorphic mouse and its development

A) Targeting construct for *Ccpg1* (*Ccpg1*^{tm1a}) as designed and used to engineer ES cells by EUCOMM/KOMP. These ES cells were obtained and used for microinjection as outlined in STAR Methods to create *Ccpg1* gene-trap founder mice. The construct results in a gene trap where the *lacZ* sequence is spliced in after exon 4. SA = splice acceptor, IRES = internal ribosome entry site, pA = poly A sequence.

B) RNA from 6 week old littermate pairs of wild-type or *Ccpg1* hypomorphic (GT/GT) mice was assayed by qRT-PCR for transcripts corresponding to enzymes analysed by mass spectrometry and/or immunoblot in main Figure 6 (n = 4 littermate pairs, ± S.E.M., *** = $p < 0.001$, two-tail t-tests). *Amy2* encodes pancreatic amylase. *Cpa1* and *Cpa2* encode the carboxypeptidases. *Prss1-3* encode isoforms of trypsinogen found in pancreatic acinar cells.

C) H and E staining of FFPE pancreas from 10 day old or 32 day old wild-type or *Ccpg1* hypomorphic mice. Scale Bar = 200 µm

D) H and E staining of FFPE, 1 day old (neonatal) wild-type or *Ccpg1* hypomorphic mice. Scale Bar = 50 µm

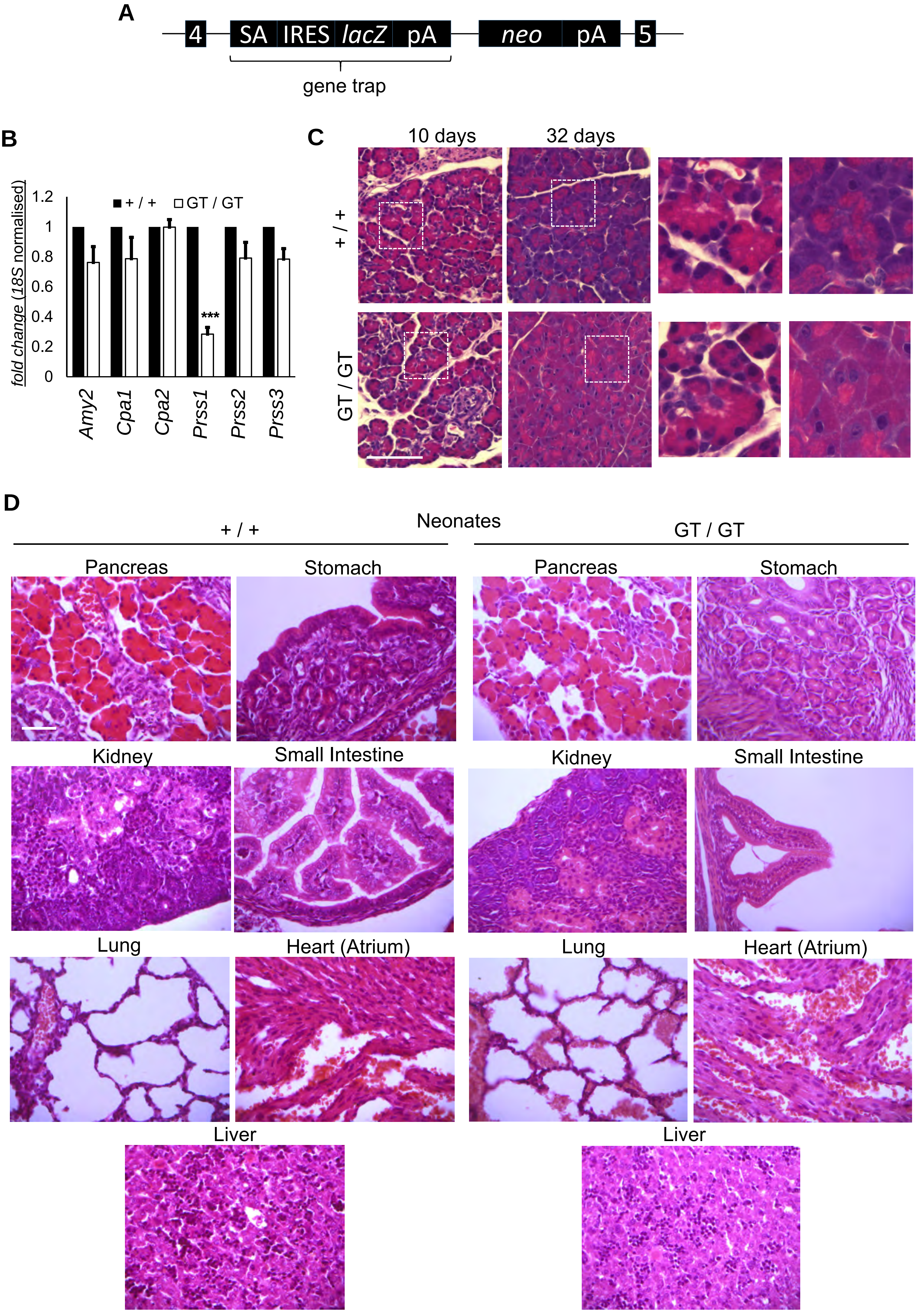

A
4
SA
IRES
lacZ
pA
neo
pA
5
gene trap
B
fold change (18S normalised)
1.2
1
0.8
0.6
0.4
0.2
0
+ / +
GT / GT
***
Amy2
Cpa1
Cpa2
Prss1
Prss2
Prss3
C
10 days
32 days
+ / +
GT / GT
D
Neonates
+ / +
GT / GT
Pancreas
Stomach
Kidney
Small Intestine
Lung
Heart (Atrium)
Liver

Supplemental Figure S6, related to Figure 7

Further histological characterisation of tissues from young adult *Ccpg1* hypomorphic mice

H and E staining of tissues from 10 week old wild-type or *Ccpg1* hypomorphic mice. Scale Bar = 50 µm

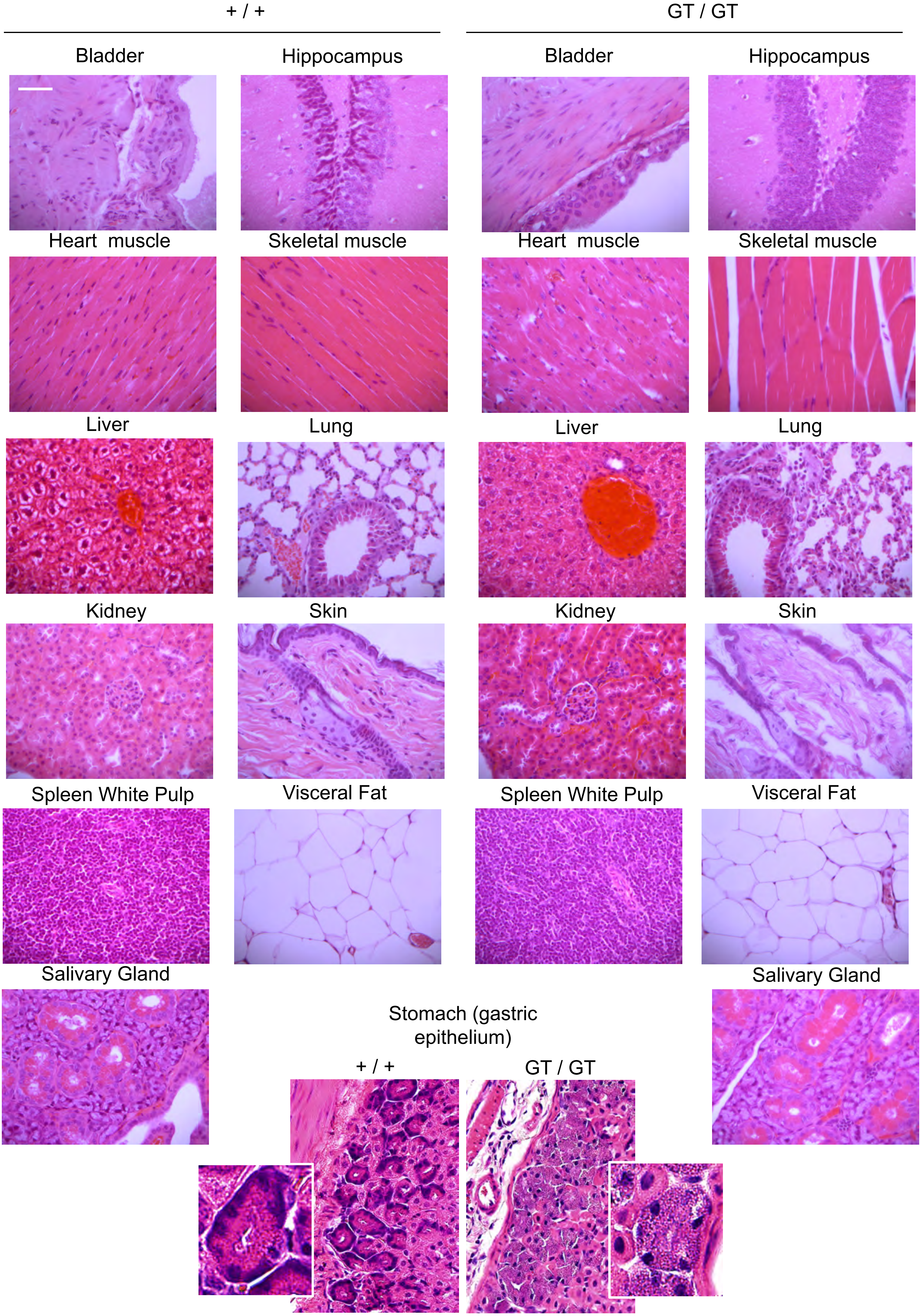
+ / +
GT / GT
Bladder
Hippocampus
Bladder
Hippocampus
Heart muscle
Skeletal muscle
Heart muscle
Skeletal muscle
Liver
Lung
Liver
Lung
Kidney
Skin
Kidney
Skin
Spleen White Pulp
Visceral Fat
Spleen White Pulp
Visceral Fat
Salivary Gland
Stomach (gastric epithelium)
+ / +
GT / GT
Salivary Gland

Supplemental Figure S7, related to Figure 7

Model for CCPG1 function

ER homeostasis involves responses to luminal stresses. A potent stress is accumulation of unfolded or insoluble protein (red stars). Transcriptional activation of effector genes that restore homeostasis is frequently part of the so-called unfolded protein response (UPR). ER stress is shown within this study to drive transcriptional activation of the gene *CCPG1*. CCPG1 is an ER-resident transmembrane (TM) protein that exposes linear peptide motifs to the cytosol, which interact with ATG8-family proteins (such as LC3, mediated via LIR motif, red bar) and FIP200 (mediated via FIR motifs, blue bars). ATG8s and FIP200 are key players in the autophagy pathway. CCPG1 protein drives ER-phagy, the selective autophagic sequestration of ER material within autophagosomes, and subsequent lysosomal degradation, via these interactions. *In vivo*, the murine pancreas requires *Ccpg1,* and presumably ER-phagy, to maintain ER homeostasis within acinar cells. In the absence of CCPG1 protein, the acinar cells accumulate ER that is laden with insoluble zymogen protein (red granules and intra-ER inclusions), resulting in elevated ER stress and a heightened UPR, and ultimately undergo cell death, a potential trigger for inflammation.

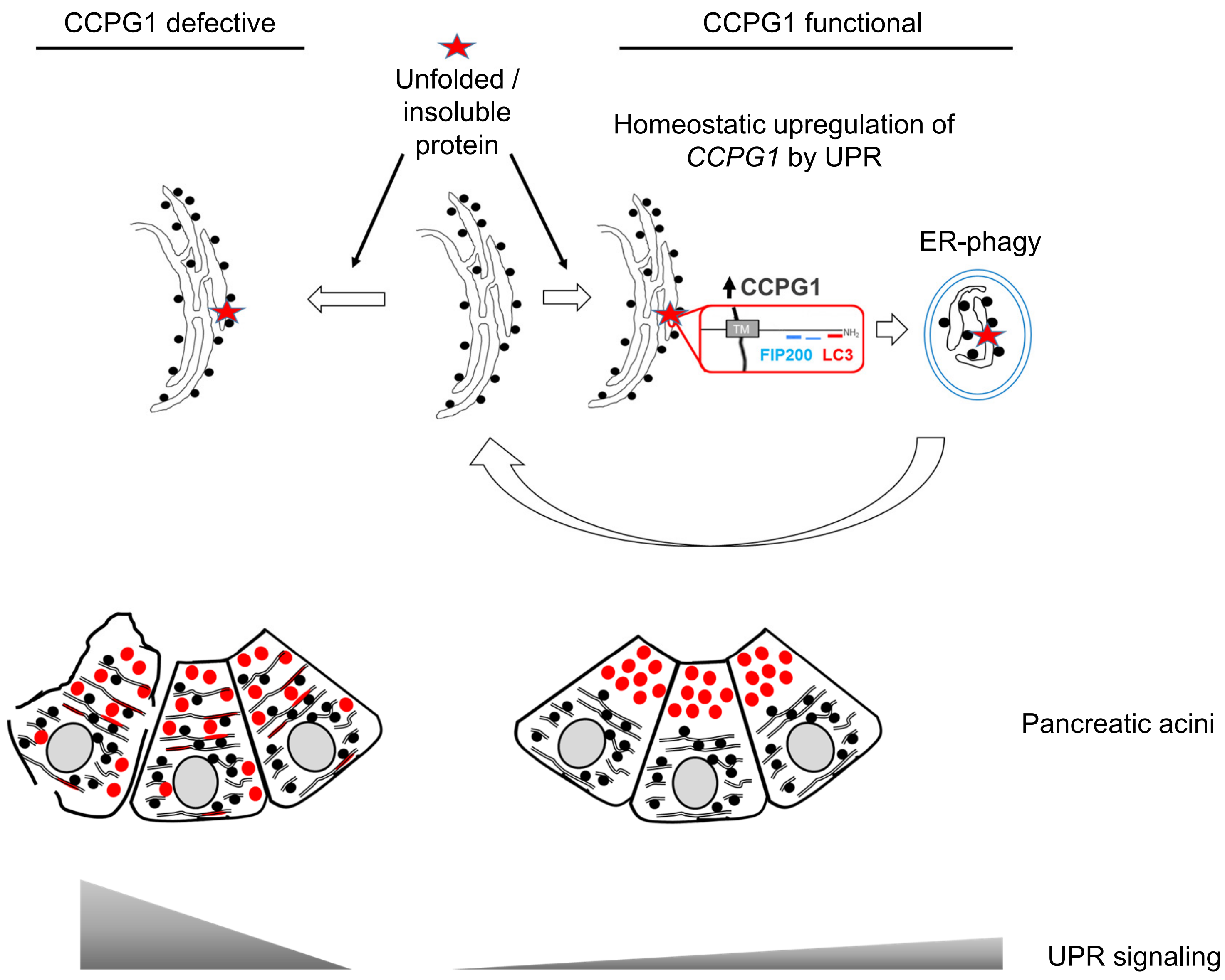

CCPG1 defective
Unfolded / insoluble protein
CCPG1 functional
Homeostatic upregulation of CCPG1 by UPR
ER-phagy
CCPG1
TM
NH2
FIP200
LC3
Pancreatic acini
UPR signaling
